# HIV-1 envelope trimer vaccine induces sex-associated differences in antibody responses: a phase 1 clinical trial

**DOI:** 10.1038/s41467-025-65101-7

**Published:** 2025-11-21

**Authors:** Emma I. M. M. Reiss, Karlijn van der Straten, Laura T. M. Graus, Marloes Grobben, Kilian E. Vlaming, Annelou I. P. van der Veen, Marinus H. Liesdek, Gabriel Ozorowski, Martin Corcoran, Hongmei Gao, Kelli M. Greene, Nicole L. Yates, Sheetal Sawant, Gius Kerster, Judith A. Burger, Stella Schonherr, Hannah M. Cheeseman, Abbey Evans, Leon R. McFarlane, Andy S. Tran, Jonathan L. Torres, Ryan N. Lin, Gyunghee Jo, Monica Tolazzi, Philipp Mundsperger, Dietmar Katinger, Albert Cupo, John P. Moore, Rob Hurks, Liffert Vogt, Maarten R. Soeters, Neeltje A. Kootstra, Gabriella Scarlatti, Georgia D. Tomaras, David C. Montefiori, Gunilla B. Karlsson Hedestam, Andrew B. Ward, Michelle Klouwens, Menno D. de Jong, Jan M. Prins, Mathieu Claireaux, Teunis B. H. Geijtenbeek, Robin J. Shattock, Marit J. van Gils, Rogier W. Sanders, Godelieve J. de Bree

**Affiliations:** 1 Department of Medical Microbiology and Infection prevention, Amsterdam UMC, location University of Amsterdam, Amsterdam, the Netherlands; 2 Amsterdam Institute for Immunology and Infectious Diseases, Amsterdam, the Netherlands; 3 Department of Experimental Immunology, Amsterdam UMC, location University of Amsterdam, Amsterdam, the Netherlands; 4 Department of Internal Medicine, Amsterdam UMC, location University of Amsterdam, Amsterdam, the Netherlands; 5 Department of Integrative Structural and Computational Biology, The Scripps Research Institute, La Jolla, CA USA; 6 Department of Microbiology, Tumor and Cell Biology, Karolinska Institutet, Stockholm, Sweden; 7 Department of Surgery, Duke University School of Medicine, Durham, NC USA; 8 Group of Mucosal Infection and Immunity, Department of Infectious Disease, Imperial College of Science, Technology and Medicine, London, UK; 9 Viral Evolution and Transmission Unit, Division of Immunology, Transplantation and Infectious Diseases, IRCCS Ospedale San Raffaele, Milan, Italy; 10 Polymun Scientific Immunbiologische Forschung GmbH, Klosterneuburg, Austria; 11 Department of Microbiology and Immunology, Weill Medical College of Cornell University, New York, NY USA; 12 Department of Radiology, Amsterdam UMC, University of Amsterdam, Amsterdam, the Netherlands; 13 Dialysis and apheresis unit, Dianet, location Amsterdam UMC, Amsterdam, the Netherlands; 14 Department of Endocrinology and Metabolism, Amsterdam UMC, University of Amsterdam, Amsterdam, the Netherlands

**Keywords:** Protein vaccines, Drug safety, Phase I trials

## Abstract

A protective vaccine will be the most powerful instrument to reduce HIV-1 infections worldwide and help bring about a lasting end to the AIDS epidemic. The single centre, randomised, open-label, uncontrolled, phase 1 ACTHIVE-001 clinical trial (NCT03961438) aims to assess the safety and immunogenicity of the ConM SOSIP.v7 native-like trimer protein vaccine, based on an HIV-1 group M consensus sequence, in HIV-negative adults. Twenty-four individuals were enrolled to receive three dosages of ConM SOSIP.v7 protein vaccine in a liposome formulation containing a high dose of the TLR4-agonist MPLA. The primary outcome is vaccine reactogenicity, whereas the main secondary outcome is binding and neutralising antibody responses. Overall, the vaccine is safe and well-tolerated. Furthermore, the vaccine elicits robust strain-specific binding and neutralising antibody responses in nearly all vaccinees. Post-hoc exploratory analyses demonstrate that female-born participants have 22- and 6-fold higher neutralisation titres after the second and third vaccination, respectively. The vaccine adjuvant induces higher levels of IL-6 secretion from in vitro cultured monocytes from female compared to male participants, providing a possible mechanistic explanation for the sex-based differences. Our study highlights the need to take sex-based differences into consideration when assessing HIV-1 vaccine candidates and adjuvants.

## Abstract (web summary)

There is still a need for effective HIV vaccines. In this phase I clinical trial, the authors show that an HIV-1 vaccine candidate, ConM SOSIP.v7, is well-tolerated in HIV-negative adults and that it elicits a strain-specific neutralising antibody response that differed between female and male participants.

## Introduction

Forty-three years after the first cases of AIDS were described and with more than one million new infections still occurring yearly, the need for an HIV-1 vaccine capable of preventing infection remains urgent. Yet the development of an effective vaccine is complicated by, amongst other things, the immense diversity of the virus^1,2^. To counter HIV-1’s diversity, many vaccine efforts focus on the induction of broadly neutralising antibodies (bNAbs) that target conserved determinants of the viral envelope glycoprotein (Env) trimer. bNAbs can protect against HIV-1 infection in passive transfer studies in both animals and humans^3–6^. They have been shown to develop in people with HIV (PWH) through a co-evolutionary process that involves exposure to multiple viral Envs over a prolonged period of time, which is why it is highly unlikely that one solitary immunogen will be capable of inducing bNAbs in vaccinees^7^. Sequential immunisation strategies are therefore being devised that involve distinct immunogens to ‘prime’, ‘shape’ and ‘polish’ the immune response towards bNAb formation^7^.

Consensus sequence-based immunogens may be suitable polishing immunogens, as a consensus sequence should contain less strain-specific antigenic determinants. This characteristic, at least in principle, should favour bNAb responses over strain-specific responses. Following the development of the prototypic stabilised BG505 SOSIP Env trimer^8^, we generated a stabilised native-like Env trimer based on a synthetic consensus of the consensus sequences of each clade in the HIV-1 Major (M) group, responsible for the majority of HIV-1 infections worldwide^9^. The resulting ConM SOSIP.v7 trimer presents all known bNAb epitopes, with the exception of the membrane-proximal external region (MPER), and is capable of inducing autologous neutralising antibody (NAb) responses in rabbits and non-human primates^9,10^. These NAb responses predominantly target the variable 1 (V1), variable 2 (V2) and variable 3 (V3) regions of the Env protein^9,10^.

In this work, we sought to establish the safety and immunogenicity of the ConM SOSIP.v7 trimer protein vaccine in humans, with a single centre, randomised, open-label, uncontrolled, phase 1 clinical trial in individuals in general good health, following an earlier experimental medicine vaccine trial^11^. To enhance immune responses against ConM SOSIP.v7, the protein was adjuvanted with a liposomal formulation composed of the Toll-like receptor 4 (TLR4) agonist monophosphoryl lipid A (MPLA), which was an efficient adjuvant for ConM SOSIP.v7 in preclinical studies^12,13^. MPLA-containing adjuvants are used in investigational adjuvant systems as well as several licensed vaccines, including AS01b used in the Shingrix (varicella-zoster) vaccine and AS04 used in the Fendrix (hepatitis B virus) and Cervarix (human papillomavirus) vaccines^14,15^.

Throughout the study, we assessed the influence of sex on the immune response against this HIV-1 vaccine candidate. Sex-based differences in immune responses to pathogens and in autoimmune pathogenesis have been documented extensively^16–18^, yet the impact of sex on vaccine-induced immune responses is less well understood, not in the least because women have historically been underrepresented in medical research^19^. Nevertheless, sex-differences have been reported for multiple antiviral vaccines such as those against influenza, hepatitis A, B, SARS-CoV-2 and yellow fever^16,20–24^. While findings vary in magnitude between types of vaccine (e.g., live attenuated or recombinant protein) and innate and adaptive responses, in general, women tend to have stronger overall immune responses than men, often leading to greater vaccine efficacy^16,17^.

As bNAbs isolated from PWH often display extensive somatic hypermutation (SHM), the current trial design also evaluated a fractional third dose aimed to promote SHM ^25^. It has been proposed that fractional dose boosting can lead to competitive antigen binding in lymph node germinal centres (GCs)^26^, thereby resulting in selection and expansion of B cells with surface immunoglobulins showing the highest antigen affinity^26^. A phase 1 study with the RTS,S/AS01_B_ malaria vaccine indeed demonstrated a significant increase in SHM and improved protection against malaria infection following a one-fifth fractional dose boost^26^. However, since the reduced dose vaccination in that particular study was also delayed compared to the full dose, it was difficult to disentangle the two variables. Mindful of this uncertainty, we implemented a comparable dosing regimen into the vaccination schedule for the current study that constitutes a fractional dose boost- and full dose-arm, but with identical intervals between vaccinations.

Here, we report on the safety and key immunogenicity outcomes of the ACTHIVE-001 clinical study (Clinicaltrials.gov identification number NCT03961438), investigating the MPLA-adjuvanted ConM SOSIP.v7 trimer protein vaccine, where we focus on the impact of successive dose reduction on B cell maturation and neutralisation endpoints, as well as the influence of sex on antibody responses and an exploration of possible factors involved. We demonstrate that the vaccine is safe and able to elicit a robust strain-specific binding and neutralising antibody response, which differs significantly between female and male participants.

## Results

### Study participants

**Fig. 1:**
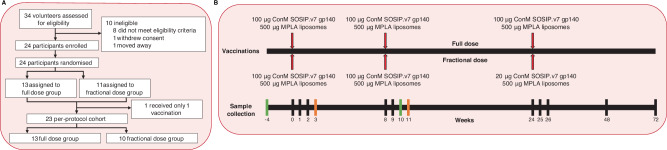
ACTHIVE-001 trial profile and vaccination schedule. **A** Modified intention-to-treat cohort: participants who received at least one vaccination (*N* = 24). Per-protocol cohort: participants who received all three vaccinations and completed at least the 48 weeks follow-up visit (*N* = 23). **B** Participants received intramuscular (i.m.) vaccinations at baseline, eight weeks and 24 weeks. Dosing between vaccine groups differs at 24 weeks, as indicated. Blood samples were collected as indicated in black. Green bars represent leukapheresis procedures. Orange bars indicate lymph node fine needle aspirations in addition to blood sampling.

Between January 2020 and November 2021, we randomly assigned 24 HIV-negative adults in general good health to receive three administrations of either full dose vaccine at baseline, eight weeks and 24 weeks, or two administrations of full dose vaccine and a one-fifth fractional dose boost at 24 weeks (Fig. 1A, B). In April 2023, 23/24 participants (96%) had completed the final week 72 visit. The median baseline age was 29 years (range 19–49) and median body-mass index (BMI) was 22.3 kg/m^2^ (range 17.1–29.0). The full dose (FD) group consisted of seven female participants (54%) and six (46%) male participants, according to sex assigned at birth. The fractional dose (FxD) group comprised seven females (64%) and four (36%) males (Table 1). One participant in the FD group was male at birth, has undergone gender-affirming surgery and currently uses feminising hormone therapy. Median age and BMI were similar between vaccine groups (median age FD group: 29 years vs. FxD group: 29 years, *p* = 0.52; median BMI FD group: 24.3 kg/m^2^ vs. FxD group: 22.0 kg/m^2^, *p* = 0.17, Mann–Whitney *U*), but different between female and male participants (median age females: 26 years vs. males: 32 years, *p* = 0.022; median BMI females: 21.8 kg/m^2^ vs. males: 24.7 kg/m^2^, *p* = 0.040, Mann–Whitney U) (Table 1).

**Table 1:** Baseline characteristics of the ACTHIVE-001 study cohort

Per vaccine group			Per sex^a^		
Age group (years)	Full dose (*N* = 13)	Fx dose (*N* = 11)	Age group (years)	Female (*N* = 14)	Male (*N* = 10)
18–25, *n* (%) ≥26–30, *n* (%) ≥31–50, *n* (%) Median per vaccine group	4 (31) 6 (46) 3 (23) 29	3 (27) 3 (27) 5 (46) 29	18–25, *n* (%) ≥26–30, *n* (%) ≥ 31–50, *n* (%) Median per sex	6 (43) 6 (43) 2 (14) 26	1 (10) 3 (30) 6 (60) 32
Sex^a^			Vaccine group		
Female, *n* (%) Male, *n* (%)	7 (54) 6 (46)	7 (64) 4 (36)	Full dose, *n* (%) Fx dose, *n* (%)	7 (50) 7 (50)	6 (60) 4 (40)
Body-mass index (kg/m²)			Body-mass index (kg/m²)		
17–25, *n* (%) ≥ 26–30, *n* (%) Median per vaccine group	9 (69) 4 (31) 24.3	9 (82) 2 (8) 22.0	17–25, *n* (%) ≥ 26–30, *n* (%) Median per sex	12 (86) 2 (14) 21.8	6 (60) 4 (40) 24.7

Data are calculated for the modified intention-to-treat cohort, which includes participants who received at least one scheduled vaccination (*N* = 24). % = Percentage of participants in each category, i.e., 100 × *n*/*N*.

^a^Sex at birth. One participant in the full dose group was male at birth, has undergone gender-affirming surgery and currently uses feminising hormone therapy. Fx dose = fractional dose.

### The adjuvanted ConM SOSIP.v7 vaccine is safe and well-tolerated in humans

In total, 294 adverse events (AEs) were reported. Most AEs were mild (Grade 1) (84.4%) to moderate (Grade 2) (12.9%), with a minority of severe events (Grade 3) (2.7%). Out of 294 AEs, 194 (66.0%) were considered related to the vaccine (adverse reactions (AR)), of which 82.0% were mild, 14.9% moderate and 3.1% severe. The most commonly reported local ARs included injection site tenderness, injection site erythema and injection site pain (Table 2), with 22/24 (91.7%) of participants reporting at least one local AR after any vaccine dose (Supplementary Table 1). The most commonly reported systemic ARs included headache, malaise and fatigue (Table 2), with 22/24 (91.7%) of participants reporting at least one systemic AR after any vaccination (Supplementary Table 1). Similar numbers of participants reported local and systemic ARs for the vaccine prime and each of the two boosts (Table 2). No serious adverse events (SAEs) or suspected unexpected serious adverse reactions (SUSARs) were reported, and none of the participants became HIV-infected or developed vaccine-induced serum positivity (VISP) for HIV.

**Table 2:** Most common local and systemic adverse reactions per vaccination

	Vaccination		
	Week 0 (*N* = 24)	Week 8 (N = 23)	Week 24 (*N* = 23)
Local adverse reactions post any vaccine dose			
Injection site tenderness, *n* (%)	16 (66.7)	16 (69.6)	15 (65.2)
Injection site erythema, *n* (%)	4 (16.7)	7 (30.4)	7 (30.4)
Injection site pain, *n* (%)	4 (16.7)	3 (13.0)	5 (21.7)
Systemic adverse reactions post any vaccine dose			
Headache, *n* (%)	6 (25.0)	7 (30.4)	6 (26.1)
Malaise, *n* (%)	7 (29.2)	5 (21.7)	5 (21.7)
Fatigue, *n* (%)	5 (20.8)	4 (17.4)	4 (17.4)

*N* = Total number of participants in the safety analysis group. *n* = Cells represent the number of participants who reported an AR in a specified category. % = Percentage of participants in each category, i.e., 100 x n/N. AEs considered possibly, probably or definitely related to the vaccine were determined ARs. Solicited ARs are reported for the full duration of the study. Source data are provided as a Source Data file.

No significant differences in number of adverse reactions (AR) were detected between the different vaccine groups (median of seven ARs per FD group participant vs. eight per FxD group participant (*p* = 0.35, Mann–Whitney *U*) (data not shown)). Similar proportions of participants per vaccine group reported ARs following any or the third vaccination (Supplementary Tables 1–2). No differences were found between the proportion of female and male participants reporting ARs throughout the study (Supplementary Table 3). There were no significant sex-based differences in the median number of ARs (females: seven vs. males: eight (*p* = 0.99, Mann–Whitney U)), nor were there significant differences when concentrating solely on local or systemic ARs (data not shown). Four participants (17%) reported at least one Grade 3 adverse event (AE) considered related to the vaccine: malaise, headache (in the same participant), fever (two participants) and injection site erythema (twice in the same participant), which all resolved spontaneously and completely (Supplementary Table 4).

One participant was withdrawn prematurely following the first vaccination after experiencing suspected vaccine-related adverse events (Fig. 1A). They reported symptoms suspect for facial angioedema after the first vaccination, however, this could not be observed by medical staff. Symptoms spontaneously resolved completely within 48 h and the participant was followed up for safety. Controlled re-challenge with adjuvanted ConM SOSIP.v7 in this participant did not result in any symptoms befitting angioedema and an allergic reaction was ruled-out by an independent consulting allergist.

In short, the adjuvanted ConM SOSIP.v7 vaccine had an acceptable safety and tolerability profile in healthy adults. A detailed evaluation of the safety profile of the vaccine regimen is presented in Supplementary Tables 1–5.

### All participants develop ConM SOSIP.v7-specific antibodies

**Fig. 2:**
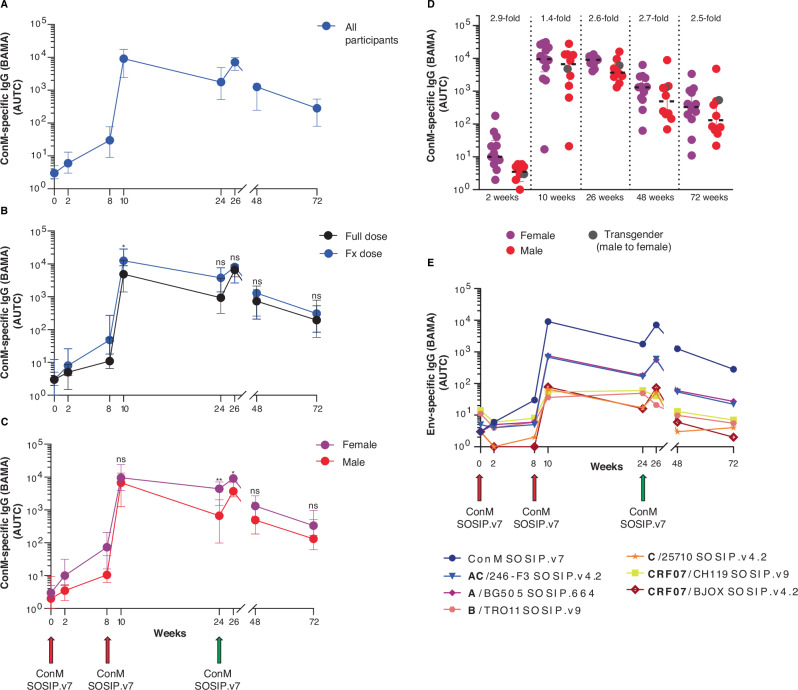
ConM SOSIP.v7-specific antibody binding (BAMA). **A** ConM SOSIP.v7-specific IgG levels measured by binding antibody multiplex assay (BAMA). Units are median fluorescence intensity (MFI). Values and error bars indicate the median and interquartile range (IQR), respectively, of the area under the median fluorescence intensity (MFI) titration curve (AUTC), calculated using trapezoidal method over the titration series at dilutions 1:50, 1:250, 1:1250, 1:6250, 1:31250, 1:156250. Values are depicted at each vaccination baseline (zero, eight and 24 weeks), two weeks post-vaccination (two, 10 and 26 weeks) and at 48 and 72 weeks. **B** ConM SOSIP.v7-specific IgG (AUTC) per vaccine group over time. Full dose group *N* = 13. Fractional (Fx) dose group *N* = 10. Values and error bars indicate the median and IQR of the AUTC. Week 10 *p* = 0.049. **C** ConM SOSIP.v7-specific IgG (AUTC) per sex at birth over time. Female *N* = 13. Male *N* = 10. Values and error bars indicate the median and IQR of the AUTC. Week 24 *p* = 0.012; week 26 *p* = 0.0065). **D** ConM SOSIP.v7-specific IgG (AUTC) per sex at birth at 2, 10, 26, 48 and 72 weeks. Values and error bars indicate the median and IQR of the AUTC. Respective fold changes are shown. Transgender individual indicated in grey. **E** Binding to a global panel of HIV-1 Env trimer proteins over time. Values indicate median AUTC, per antigen. All figures represent the per-protocol cohort (*N* = 23). Green arrows signify difference in vaccine dose at 24 weeks. Differences between groups and sex were calculated by a two-tailed Mann–Whitney U-test. *p < 0.05, **p < 0.01, ns = not significant. Source data are provided as a Source Data file.

After three vaccinations, all per-protocol vaccine recipients (23/23) produced serum binding antibodies to the ConM SOSIP.v7 antigen. Median antigen-specific IgG levels quantified by binding antibody multiplex assay (BAMA) varied between 9030 (median of the area under the titration curve (AUTC)) at 10 weeks (range of AUTC 17–31,259) and 7121 at 26 weeks (range of AUTC 1312–16,101) (Fig. 2A). The levels of antigen-specific IgG differed significantly between the FD and FxD vaccination groups at 10 weeks, prior to divergence of the administered vaccine dose (median AUTC FD group: 4839 vs. FxD group: 12,447, *p* = 0.049, Mann–Whitney *U*), but no longer at 26 weeks and beyond (Fig. 2B). Substantial and statistically significant differences were observed between females and males on the day of and after the third vaccination (Fig. 2C, D) (24 weeks: median AUTC females: 4412 vs. males: 662, *p* = 0.0065, Mann–Whitney *U*) (26 weeks: median AUTC females: 9034 vs. males: 3675, *p* = 0.012, Mann–Whitney *U*). Antigen-specific IgG decreased over time with a 6-fold decline between 26 weeks (median AUTC 7121) and 48 weeks (median AUTC 1255), followed by a further 5-fold decrease at 72 weeks (median AUTC 282) (Fig. 2A). Previously observed differences between sexes were no longer statistically significant at 48 and 72 weeks (Fig. 2C, D). These findings were corroborated by enzyme-linked immunosorbent assay (ELISA) (Supplementary Fig. 1a–d) and were highly comparable (Spearman *r* = 0.77, *p* < 0.0001) (Supplementary Fig. 2a). Additionally, sera from nearly all participants showed modest but broad cross-reactivity to a panel of HIV-1 Env proteins reflecting global strains at 26 weeks. Sera from 92% of participants cross-reacted with 6/6 viral strains, while the remaining sera cross-reacted with four or five strains (Fig. 2E).

**Fig. 3:**
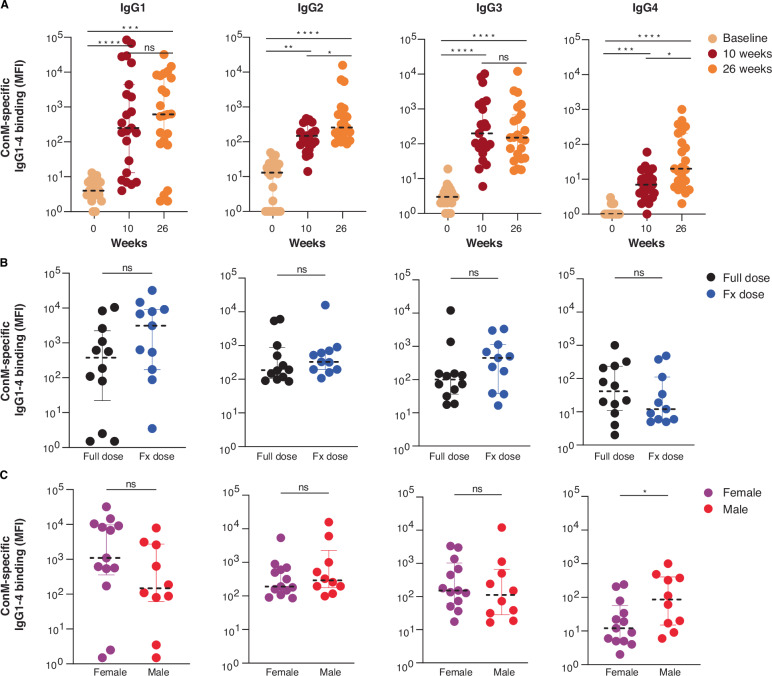
ConM SOSIP.v7-specific IgG subtypes. ConM SOSIP.v7-specific IgG1-4 binding as measured by Luminex immunoassay and presented as blank-corrected median fluorescence intensity (MFI) (respective serum dilutions IgG1 1:50,000; IgG2 1:100; IgG3 1:500; IgG4 1:100). Values and error bars for each figure indicate the median MFI and interquartile range (IQR). **A** ConM SOSIP.v7-specific IgG1-4 binding (MFI) at baseline, 10 and 26 weeks. IgG1 week 0 vs. 26 *p* = 0.0001; IgG2 week 0 vs. 10 *p* = 0.0012; IgG2 week 10 vs. 26 *p* = 0.024; IgG4 week 0 vs. 10 *p* = 0.0005; IgG4 week 10 vs. 26 *p* = 0.045. **B** ConM SOSIP.v7-specific IgG1-4 binding (MFI) per vaccine group at 26 weeks. Full dose group *N* = 13. Fractional (Fx) dose group *N* = 10. **C** ConM SOSIP.v7-specific IgG1-4 binding (MFI) per sex at birth at 26 weeks. Female *N* = 13. Male *N* = 10. IgG4 females vs. males *p* = 0.040. All figures represent the per-protocol cohort (*N* = 23). A Friedman test followed by a Dunn’s multiple comparison test was used for comparing three time points for the same individuals. Differences between groups and sex were calculated by a two-tailed Mann–Whitney *U*-test. *p < 0.05, **p < 0.01, ***p < 0.001, ****p < 0.0001, ns = not significant. Source data are provided as a Source Data file.

To specify the contribution of different IgG subclasses to the antigen-specific serum response, we measured IgG1-4 levels using a custom multiplex assay (Luminex). Nearly all participants who received the complete vaccine regimen developed detectable serum levels of antigen-specific IgG subtypes 1–4. Generally, the magnitude of the response increased after each consecutive boost. Between 10 and 26 weeks the response increased for IgG1 (3-fold), IgG2 (2-fold) and IgG4 (3-fold), while it remained stable for IgG3 (Fig. 3A). FxD recipients had higher levels of antigen-specific IgG1 and IgG3 compared to FD recipients at 26 weeks, yet these differences were not statistically significant (Fig. 3B) and were already present at 10 weeks (not shown). Differences between female and male vaccine recipients were found for IgG1 and IgG4. Females developed 7-fold higher antigen-specific IgG1 responses, although the difference was not statistically significant (*p* = 0.12, Mann–Whitney *U*), while males produced 7-fold higher levels of IgG4 (*p* = 0.04, Mann–Whitney *U*) (Fig. 3C) at 26 weeks. In summary, the adjuvanted ConM SOSIP.v7 protein vaccine was capable of eliciting antigen-specific IgG responses in all vaccine recipients and cross-binding to several other Env proteins representing globally circulating HIV-1 strains, with differences between female and male vaccinees in both total vaccine-specific IgG and IgG subtypes.

### Females develop stronger autologous neutralising antibody responses

**Fig. 4:**
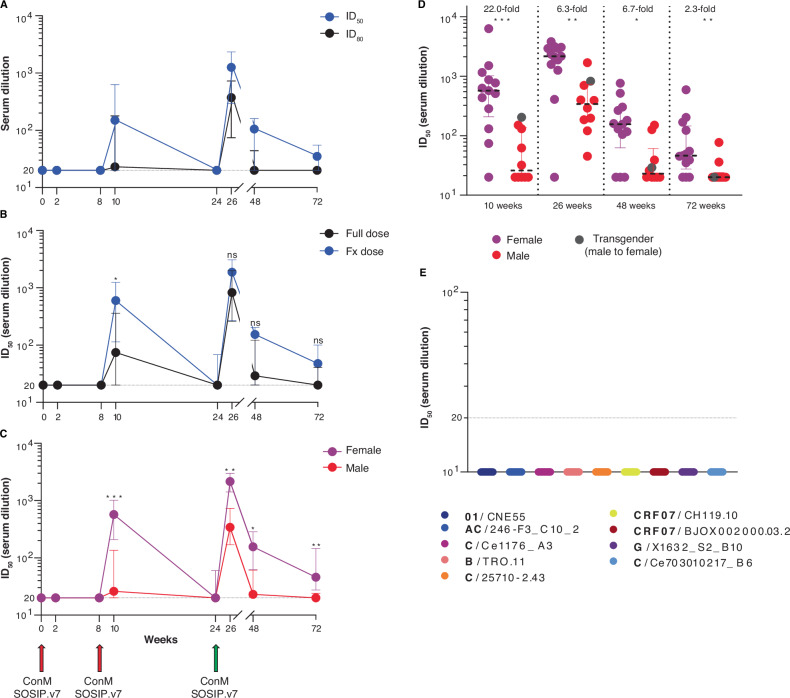
Pseudovirus serum neutralisation over time. **A** ConM-pseudovirus serum neutralisation at each vaccination baseline (zero, eight and 24 weeks), two weeks post-vaccination (two, 10 and 26 weeks) and at 48 and 72 weeks. Values are the serum dilution at which relative luminescence units (RLUs) were reduced 50% (ID_50_) or 80% (ID_80_) compared to virus control wells. Values and error bars indicate the median ID_50_ or ID_80_ and interquartile range (IQR). **B** ConM-pseudovirus serum neutralisation ID_50_ values per vaccine group over time. Values and error bars indicate the median ID_50_ and IQR. Full dose group *N* = 13. Fractional (Fx) dose group *N* = 10. Week 10 *p* = 0.33. **C** ConM-pseudovirus serum neutralisation ID_50_ values per sex at birth over time. Values and error bars indicate the median ID_50_ and IQR. Female *N* = 13. Male *N* = 10. Week 10 *p* = 0.0006; week 26 *p* = 0.0011; week 48 *p* = 0.016; week 72 *p* = 0.0077. **D** ConM-pseudovirus serum neutralisation ID_50_ values per sex at birth at 10, 26, 48 and 72 weeks. Values and error bars indicate the median ID_50_ and IQR. Respective fold changes are shown. Transgender individual indicated in grey. **E** Week 26 sera were tested against a panel representative of global HIV-1 varieties, as indicated. All figures represent the per-protocol cohort (*N* = 23). Green arrows signify difference in vaccine dose at 24 weeks. Differences between groups and sex were calculated by a two-tailed Mann–Whitney *U*-test. *p < 0.05, **p < 0.01, ***p < 0.001, ns = not significant. Source data are provided as a Source Data file.

Nearly all (22/23) per-protocol vaccine recipients developed NAbs against the autologous neutralisation-sensitive ConM virus following the third vaccination, with half maximum inhibitor dilution (ID_50_) values ranging from 45 to 3817 at 26 weeks (median ID_50_ 1417) (Fig. 4A). A weaker response (median ID_50_ 437, range 32–6276) could already be detected in the majority (17/23) of participants two weeks after the second vaccination (Fig. 4A). Serum neutralisation decreased in all initially responsive participants to a median ID_50_ of 111 (range 20–759) and 36 (range 20–591) at 48 and 72 weeks, respectively, representing a 13-fold decline over the first five months (26 to 48 weeks) and a 3-fold decline over the next five and a half months (48 to 72 weeks) (Fig. 4A). Autologous serum neutralisation correlated to antigen-specific IgG titres as quantified by both BAMA (Spearman *r* = 0.860, *p* < 0.0001) and ELISA (Spearman *r* = 0.575, *p* = 0.0041) (Supplementary Fig. 2b, c). IgG3 also correlated positively with autologous serum neutralisation at 26 weeks (Spearman *r* = 0.450, *p* = 0.03) (Supplementary Fig. 3c), while IgG4 levels showed a negative correlation, albeit not statistically significant (Spearman *r* = −0.303, *p* = 0.16) Supplementary Fig. 3d).

Although FxD vaccinees tended to have stronger serum neutralising responses at 26 weeks (median ID_50_ FD group: 823 vs. FxD group: 1867), these differences were already observed prior to the fractional dose boost and were not statistically significant (*p* = 0.28, Mann–Whitney *U*) (Fig. 4B). Similar to binding antibodies, significant differences in neutralisation were most likely linked to sex. Female participants overall developed more potent autologous neutralisation over time, culminating in a 22-fold difference at 10 weeks (median ID_50_ females: 571 vs. males: 26, *p* < 0.001, Mann–Whitney *U*) and 6-fold difference at 26 weeks (median ID_50_ females 2156 vs. males 341, *p* < 0.001, Mann–Whitney *U*) (Fig. 4C, D). Differences between sexes were also observed within vaccine groups (Supplementary Fig. 4a). No significant differences between vaccine groups were found over time (Fig. 4B), yet the sex-based differences remained statistically significant at both 48 weeks (median ID_50_ females: 156 vs. males: 23, *p* = 0.016, Mann–Whitney *U*) and 72 weeks (median ID_50_ females: 46 vs. males: 20, *p* = 0.016, Mann–Whitney *U*) (Fig. 4C, D). Modest neutralisation of the neutralisation-sensitive (Tier 1b) ConS virus was detected after the full vaccine regimen at 26 weeks (data not shown), but not of a virus panel consisting of neutralisation-resistant (Tier 2) viruses, representing global HIV-1 diversity (Fig. 4E). In short, the adjuvanted ConM SOSIP.v7 vaccine elicited a potent autologous NAb response in nearly all recipients. No differences were associated with the fractional dose but NAb responses varied significantly between female and male vaccinees.

### Antibody responses map to V1V2 apex, CD4 binding site and trimer base

**Fig. 5:**
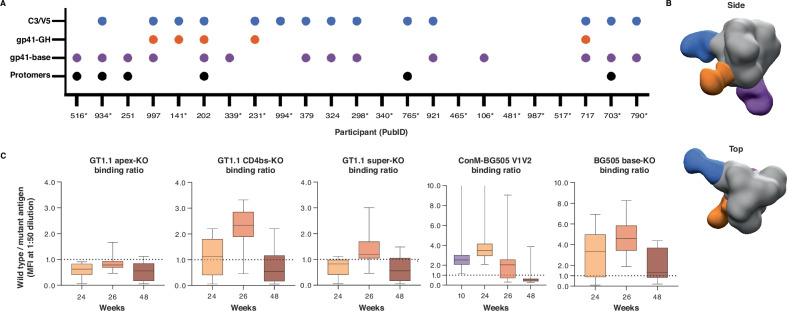
Antibody specificities. **A** Summary of Env epitopes targeted by polyclonal antibodies from each participant at 26 weeks as determined by EMPEM. Protomer designates that antibody-induced trimer dissociation into monomers was observed and does not imply that the antibodies are monomer-specific. For samples with an asterisk (*), additional Fab binding was observed in 2D classification but epitope mapping in 3D was unsuccessful. gp41-GH: gp41-glycan hole. gp41-base: gp41 trimer base. **B** Composite figures of the various antibody specificities detected by the 3D analysis are shown (side and top views), based on EMPEM data for PubID 202. **C** Binding ratio of wild type antigen (BG505 SOSIP.GT1.1) to mutant as determined by binding antibody multiplex assay (BAMA) (median fluorescence intensity (MFI): BG505 SOSIP.GT1.1 to BG505 SOSIPv8.1-GT1.1 apex-KO, BG505 SOSIPv4.1-GT1.1 CD4bs-KO and BG505 SOSIPv8-GT1.1 super-KO (apex and CD4bs epitope KO mutations combined); BG505 SOSIP.664 to ConM-BG505 V1V2 and BG505 SOSIP.v5 base-KO + 613 T. Serum samples from all 23 per-protocol participants were tested at each time point. Samples were tested in replicates and a mean of two replicates was reported. Wild type MFI at a 1:50 dilution was divided by mutant MFI at similar dilution for final calculations. Box plots indicate median values, 25^th^ and 75^th^ percentiles; whiskers represent minima and maxima. Source data are provided as a Source Data file.

We mapped the dominant specificities of the vaccine-induced serum antibodies by using electron microscopy-based polyclonal epitope mapping (EMPEM)^27^. The majority of the vaccinees developed antibodies against the trimer base (gp41-base). Such a response is consistently observed in vaccination studies with the prototypic BG505 SOSIP trimer and is a consequence of the soluble trimer design, which creates a large glycan-free protein surface at the bottom of the trimer where the viral membrane would normally be^28^. Several participants developed antibodies against putative glycan holes on gp41 (gp41-GH) and/or antibodies that cause trimer disassembly into monomeric gp140 (protomers). Both types of responses have also been observed following immunisation with BG505 SOSIP trimers in humans^28^, with antibody-induced trimer disassembly common after repeat immunisations using the same immunogen^29,30^. Responses to the gp41-base or putative glycan holes have not typically correlated with neutralisation, but the human bNAb 3BC315 has been reported to bind gp41 and disassemble Env trimers^31^, suggesting that the antibody-induced trimer disassembly (protomers) observed by EMPEM could also contribute to the ConM-neutralisation. The most notable response in this study was directed to the C3/V5 region (Fig. 5A, B). This response had a different binding angle than C3/V5 responses induced by the BG505 SOSIP protein^8^ and may represent a ConM SOSIP.v7-specific C3/V5 response, as also observed in non-human primates^10^. Notably, the ConM sequence lacks a potential N-linked glycosylation site (PNGS) at position 363 and also has shorter V5 loop compared to BG505 (Supplementary Fig. 5a).

In order to map subdominant serum antibody specificities, we measured serum IgG responses against a panel of HIV-1 trimer antigens containing specific epitope knock-outs (KO). This panel included the recombinant ConM-BG505 V1V2 protein^9,10^, consisting of ConM SOSIP.v7 displaying the BG505 V1V2 domain, wild-type BG505 SOSIP^8^, the germline targeting variant BG505 SOSIP.v8.1-GT1.1 (GT1.1)^32^, and a series of epitope KO trimers. Each of these KO trimers allows for specification of antibodies able to bind to certain previously studied epitopes such as the trimer base (BG505 SOSIP.v5 base-KO + 613 T), apex (BG505 SOSIP.v8.1-GT1.1 apex-KO), CD4 binding site (CD4bs) (BG505 SOSIP.v4.1-GT1.1 CD4bs-KO) and a combination of the latter two (BG505 SOSIP.v8-GT1.1 super-KO, combining the apex and CD4bs epitope KO mutations). As shown in Fig. 2E, all week 26 sera were positive for binding to the clade A BG505 SOSIP^8^ trimer and the germline targeting GT1.1 trimer (data not shown). Even though binding to these Env trimers was notably weaker than binding to autologous ConM SOSIP.v7 protein, relative binding to the respective (KO) mutants provided an insight into which epitopes were likely targeted. The differential binding to GT1.1 versus its CD4bs-KO variant revealed that CD4bs-targeting antibodies were present particularly after the second boost, at week 26. A strong differential binding was observed with the ConM SOSIP.v7 trimer versus the ConM-BG505 V1V2 trimer, pointing at the presence of V1V2 apex-specific antibodies, and/or antibodies that were dependent on the presence of the ConM V1V2 domain, which was the most dominant target of neutralising antibodies in non-human primates^10^ (Fig. 5A–C). Vaccination with the soluble ConM SOSIP.v7 trimer protein also elicited an off-target trimer base-specific antibody response, i.e., a response not typically associated with neutralisation, consistent with the EMPEM observations (Fig. 5A–C).

We also performed serum neutralisation depletion experiments similar to those described by Sliepen et al., using the ConM SOSIP.v7 and chimeric ConM-BG505 V1V2 Env trimers^9,10^. While the ConM SOSIP.v7 trimer itself depleted the majority of neutralisation activity in nearly all participant sera, the addition of ConM-BG505 V1V2 showed notable levels of depletion in approximately half of the sera, suggesting that (1) NAbs that are dependent on the ConM V1V2 were present in half of the sera and (2) other autologous neutralisation epitopes than the V1V2 region were targeted in the remaining individuals (Supplementary Fig. 5b). In short, through the analyses described above, we identified antigen-specific serum antibodies directed to the V1V2 region on the trimer apex, CD4bs and an off-target response to the trimer base. No differences between female and male vaccinees were found within the EMPEM or neutralisation depletion assays (data not shown), yet subtle differences were seen in the KO mutant binding analyses. Female participants developed a CD4bs-directed response already at 24 weeks (WT/mutant binding ratio females: 1.7 vs. males: 0.4, *p* = 0.074), with male participants catching up at 26 weeks (WT/mutant binding ratio females: 2.5 vs. males: 2.0, *p* = 0.088) (Supplementary Fig. 5c).

### Males display higher SHM levels in ConM SOSIP.v7-specific memory B cells

Next, we sorted antigen-specific memory B cells using the fluorescence-activated cell sorting (FACS) strategy shown in Supplementary Fig. 6a. Memory B-cells were defined either by unbiased cell annotation based on transcriptomics or the presence of SHM. The frequency of antigen-specific memory B cells within the total memory B cell population was 0.41% (range 0.082–1.62%) at 26 weeks and 0.19% (range 0.064–0.38%) at 48 weeks (Supplementary Fig. 6b, c), but similar between sexes and vaccine groups at either time point (Supplementary Fig. 6d–g).

To assess SHM levels in BCR sequences of class-switched memory B cells, we performed high-throughput single-cell sequencing (10X Genomics®) post-immunisation at 26 and 48 weeks. We also generated individualised germline immunoglobulin heavy chain variable (IGHV) gene databases for each participant by deep sequencing pre-vaccination PBMCs and applying IgDiscover^33^. This enabled highly precise germline IGHV gene assignments, determination of SHM levels and definition of CDRH3 lengths, allowing us to make comparisons between the naive repertoire and mature antigen-specific BCR sequences.

**Fig. 6:**
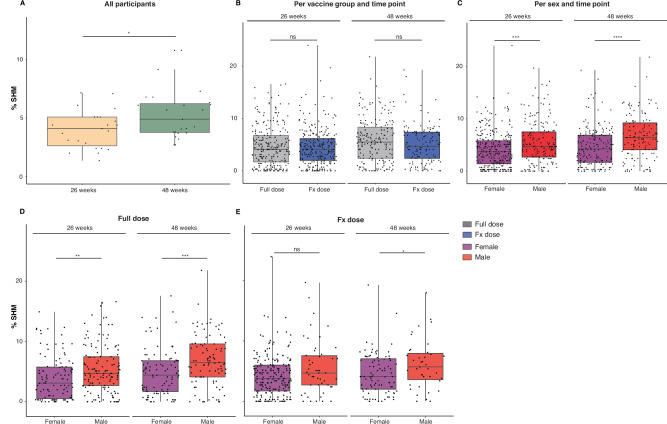
Somatic hypermutation levels in ConM SOSIP.v7-specific memory B cells. V-gene somatic hypermutation (SHM) levels (%) within the ConM SOSIP.v7-specific memory B-cell population, measured at 26 weeks and 48 weeks for **A** the complete (*N* = 23) per-protocol cohort (*p* = 0.048), **B** the Full dose group (*N* = 13) and Fractional (Fx) dose group (*N* = 10), **C** female (*N* = 13) and male (*N* = 10) participants (week 26 *p* = 0.00086; week 48 *p* = 1.7 × 10^−5^), **D**, **E** female and male participants per vaccine group, per time point. Full dose female (*N* = 7), Full dose male (*N* = 6), Fx dose female (*N* = 6), Fx dose male (*N* = 4) (**D** week 26 *p* = 0.0023; week 48 *p* = 0.00045; **E** week 48 *p* = 0.042). Box plots indicate median SHM values, 25^th^ and 75^th^ percentiles; whiskers represent minima and maxima. Differences between groups and sex were calculated by two-sided Wilcoxon signed rank test, paired comparisons were calculated by donor median SHM two-sided paired Wilcoxon signed rank test. *p < 0.05, **p < 0.01, ***p < 0.001, ****p < 0.0001, ns = not significant (*p* > 0.05). Source data are provided as a Source Data file. Sequence data were uploaded to a public repository (see ‘Data Availability’).

For the complete per-protocol cohort, the median IGHV gene nucleotide SHM level within the antigen-specific memory B cell population was 4.1% at 26 weeks and increased to 5.3% at 48 weeks (*p* = 0.048) (Fig. 6A, Supplementary Fig. 6h). An increase in SHM over time without further vaccination is consistent with observations made after COVID-19 infection^34,35^. Absolute SHM values did not differ between vaccine groups at either 26 weeks (FD: 4.1%, FxD: 4.1%, *p* = 0.71) or 48 weeks (FD: 5.5%, FxD: 4.7%, *p* = 0.13) (Fig. 6B). SHM levels were, however, significantly different between female and male vaccinees at 26 weeks (females: 3.7%, males: 4.7%, *p* = 0.0009), and following a particularly steep increase in males, this difference became more pronounced at 48 weeks (females: 4.3%, males: 6.4%, *p* < 0.0001) (Fig. 6C–E, Supplementary Fig. 6i, j).

Overall, we noted that the majority of mature BCR sequences (at weeks 26 and 48 taken together) utilised IGHV-genes from the IGVH3 family (57%), representing an enrichment compared to the pre-vaccination repertoire (45%). Other IGHV family usage remained more or less consistent between baseline and post-vaccination (Supplementary Fig. 7a, b). Subtle differences in IGHV family frequencies were seen between females and males (Supplementary Fig. 7c). While IGHV3 genes dominated in males, the IGHV usage was more diverse in females and included IGVH2, −5 and −7, genes rarely observed in the ConM SOSIP.v7-specific repertoire of males. The average CDRH3 length for antigen-specific memory B cells was 15.1 amino acids (aa), which is very similar to the average CDRH3 length in humans (14.8 aa^36^). This length did not change significantly over time and was similar between dose groups and sexes (Supplementary Fig. 7d–f).

### Testosterone levels correlate inversely with autologous neutralisation

To gain additional insights into the observed sex-differences in serological outcomes, we assessed levels of female and male sex steroid hormones. Plasma levels of oestradiol, progesterone and testosterone were measured through routine clinical laboratory facilities prior to each vaccination at baseline, eight and 24 weeks. Correlations between hormone levels and autologous neutralisation, antigen-specific IgG and IgG1-4 at 10 and 26 weeks were considered. For the complete per-protocol cohort taken together, testosterone levels correlated inversely and significantly with autologous neutralisation titres (data not shown). However, this effect was attributable to evident biological differences between female and male-born participants. Within the female and male groups no correlations were found between any hormone and autologous serum neutralisation (data not shown). We did observe inverse correlations within the female and male groups between testosterone and IgG3 levels, and oestradiol and IgG3 levels, but these correlations did not reach statistical significance after Bonferroni multiplicity correction (threshold *p* < 0.004) (Supplementary Fig. 8a–g).

### Female-derived PBMCs secrete more IL-6 in response to the vaccine

**Fig. 7:**
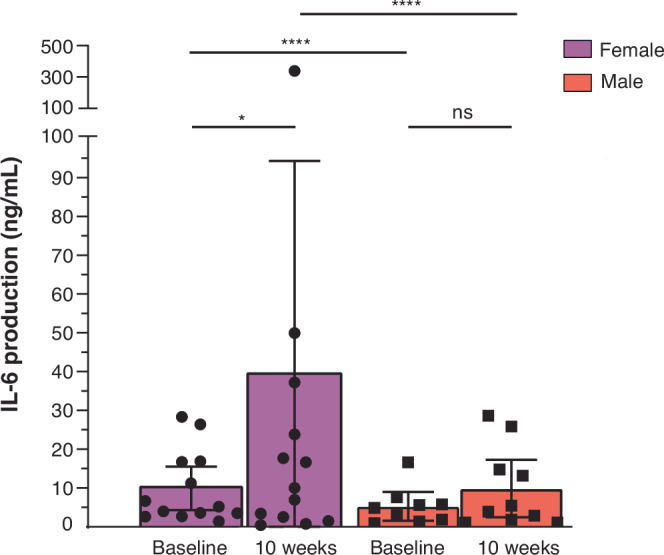
IL-6 secretion by PBMCs following ConM SOSIP.v7/MPLA stimulation. PBMCs were incubated for 24 h with ConM SOSIP.v7 (5 µg/mL) and MPLA (30 µg/mL). Subsequently, supernatant was removed and IL-6 levels were determined using ELISA. Data is visualised for all participants, comprising female (*N* = 13 at both time points) and male study participants (*N *= 9 at baseline due to missing sample, *N* = 10 at 10 weeks). Box plots indicate median values, 25^th^ and 75^th^ percentiles; whiskers represent minima and maxima. Statistical testing was performed between both groups using repeated measures two-way NOVA followed by a Tukey multiple comparisons test. *p = 0.030, ****p < 0.0001, ns = not significant. Source data are provided as a Source Data file.

Next, we investigated the innate immune responses between female and male study participants. PBMCs isolated at baseline (−4 weeks) and after the second vaccination (10 weeks) were stimulated in vitro with the complete vaccine, the ConM SOSIP.v7 trimer protein and the MPLA adjuvant separately, and control TLR agonists. While no differences were observed in the secretion of IL-1β, IL-10, or IL-12p70 (data not shown), we noted statistically significant differences between female and male participants in their PBMC’s capacity to secrete IL-6, which is involved in B cell maturation and antibody responses^37,38^. At baseline, PBMCs from female participants demonstrated a 1.9-fold higher induction of IL-6 compared to male participants in response to the vaccine (ConM SOSIP.v7 plus MPLA; *p* < 0.0001). The IL-6 induction from PBMCs was substantially more pronounced at week 10, suggestive of trained immunity following the vaccination at week 8^39–41^. At that time-point IL-6 secretion from female PBMCs was 4-fold higher than from male samples (*p* < 0.0001) (Fig. 7). In contrast to the ConM SOSIP.v7 trimer protein alone, TLR4 agonist MPLA alone induced IL-6 secretion from PBMCs (Supplementary Fig. 9a, b), implying that the adjuvant was responsible for the IL-6 induction. Similar results were obtained when non-responder samples, defined by lacking the capacity to upregulate IL-6 in response to LPS, were excluded from the analyses (Supplementary Fig. 9c). Since females have been shown to express higher levels of TLR4 on certain immune cells, we investigated whether this was the case for our study cohort, potentially contributing to more robust TLR4 activation. TLR4 expression levels on monocytes were determined by flowcytometry, as well as TLR4 mRNA expression levels relative to a selected housekeeping gene (GAPDH). Neither of these analyses, however, demonstrated significant differences in TLR expression levels between female and male participants at baseline (week −4) (Supplementary Fig. 10).

## Discussion

A protective HIV-1 vaccination regimen should elicit antibodies capable of neutralising most circulating strains. Replicating the development of bNAbs during natural HIV-1 infection is thought to require multiple different immunogens given in sequence, referred to as ‘priming’, ‘shaping’, and ‘polishing’ immunogens^7^. Our study is one of several recent efforts testing novel immunogens aimed to contribute to inducing bNAbs^11,28,42^. These efforts include studying priming immunogens, such as germline-targeting immunogens that are specifically designed to activate B cells expressing precursor bNAb BCRs^32,42^. Here, we clinically evaluated a stabilised native-like Env trimer based on an artificial consensus sequence with characteristics that might favour bNAb responses over strain-specific responses. Given that there were no expected safety concerns for the Env-vaccine component and that potential reactogenicity of the MPLA adjuvant was considered acceptable based on large-scale previous studies^43–45^, an open-label study design was chosen. We have shown that the adjuvanted ConM SOSIP.v7 trimer protein vaccine had an acceptable safety and tolerability profile in HIV-negative adults in good general health. Reported reactogenicities were in line with previous clinical reports with MPL-adjuvanted vaccines^43–45^. Furthermore, the adjuvanted ConM SOSIP.v7 vaccine was capable of eliciting antibody responses that were cross-reactive with Env proteins from diverse globally circulating HIV-1 strains. We propose that ConM SOSIP.v7 might be a suitable ‘polishing’ immunogen, following vaccination with germline-targeting ‘priming’ and ‘shaping’ immunogens.

A main finding of our study was that female participants raised higher autologous neutralising antibody responses to the neutralisation-sensitive ConM strain than males. Biological differences between females and males are a known source of variation in the immune response to vaccination. There are some studies that reported on (sex-differences in) antibody neutralisation in seasonal Influenza and SARS-CoV-2 vaccination, yet the effect size in these studies was generally limited^21,24^. For example, differences in neutralisation were indeed found benefiting female recipients of the BNT162b2 SARS-CoV-2 vaccine, yet the maximum fold-difference was approximately 1.5^24^. We therefore argue that the magnitude of the differences found in our cohort was remarkable, especially in the context of HIV-1 vaccines. Interestingly, these differences were not limited to the quantity of the antibody response, but were also evident when studying the functionality of the response and demonstrated by the striking 22-fold and 6-fold differences in autologous NAb titres at 10 and 26 weeks, respectively. To our knowledge, a finding of similar magnitude has not yet been reported in preclinical or clinical HIV vaccine studies. In addition, we identified differences in IgG subtypes (higher IgG1 and lower IgG4 levels in females compared to males), suggesting that the female antibody response may consist of more (Fc-)functional antibodies and therefore could be more capable of inducing antibody-mediated effector functions. Indeed, another study focusing primarily on Fc effector functionality corroborated these findings, likewise suggesting qualitative differences between the female and male antibody response to ConM SOSIP.v7 protein^46^, in addition to a quantitative difference.

The contribution of sex hormones to the sex-differences in immunity was notable, yet the reported data alone are insufficient to point at a causal relationship between hormones and antibody responses in our cohort. This was true for testosterone in particular, where findings were consistent with biological differences between the sexes and did not prove a causal relation. Larger follow-up studies also considering hormonal fluctuations, for example, during the menstrual cycle, use of contraception and age-related changes, and their direct impact on antibody response could provide a more nuanced understanding of these differences. Anecdotally, the transgender participant (male to female) may be illustrative for the relative influence of both hormones and genetics: while considered a low to average responder within the female cohort, this individual was one of the top responders when clustered with male-born participants.

Sex-differences in TLR receptor expression and signalling have been described, in particular for TLR4 and the X-chromosome linked TLR7 and TLR8^19,47^, which have for example been linked to lower viremia during acute HIV-infection in females^47^. We observed that female-derived PBMC exhibited a significantly stronger IL-6 response following stimulation with the TLR4 agonist MPLA, both alone and in combination with the ConM SOSIP.v7 trimer protein. As the significance seems to be driven by a limited number of participants, it will be useful to confirm these findings in larger cohorts, using monocytes from females at different stages of the menstrual cycle and titrating MPLA (for example, in studies using AS01b adjuvant in combination with HIV-1 Env trimers (e.g., ClinicalTrials.gov identification numbers NCT03699241, NCT04224701)). We note that in our study the MPLA content per vaccine dose (500 µg), was 10 times higher than that in AS04 and AS01b (50 µg) and 20 times higher than that in AS01e (25 µg)^14^, potentially explaining why such large differences between sexes have not been observed in previous studies with these adjuvants.

In contrast to the serological responses, which were stronger in females, SHM levels in ConM SOSIP.v7-specific memory B cells were substantially higher in males, in particular at week 48, i.e., six months following the last vaccination (6.4% compared to 4.3%, *p* < 0.0001). We are not aware of any studies reporting differences in SHM levels between females and males and we do not have a satisfactory explanation. However, we do note that SHM and antibody titre do not need to be linked. SHM is mostly a consequence of a strong germinal centre (GC) reaction, while antibody titres, in particular at peak immunity, are mediated to a large extent by short lived plasmablasts, not necessarily cells that are a late product of a strong GC reaction. It could therefore be that males have a longer and/or stronger GC response, which may explain why at earlier timepoints female participants perform better and males catch up at later timepoints based on serum data. Furthermore, males also had higher antigen-specific IgG4 levels than females, indicative of more progressive class-switch recombination (CSR) in males. Since SHM and CSR are both regulated by Activation-induced cytidine deaminase (AID)^48^, the observations might suggest that AID is more active in males in response to the MPLA adjuvanted ConM SOSIP.v7 trimer protein vaccine. Additionally, T follicular helper (Tfh) cell responses could also contribute to the observed sex-differences. Further exploration is needed for a better understanding of these contributors.

Characteristics such as BMI and age are known to play a role in the development of immune responses and differed significantly between females and males at baseline, thereby acting as potential confounding factors. Additionally, the sex-differences in (neutralising) antibody responses described above were based on post-hoc exploratory analyses and not pre-determined as primary or secondary study objectives. However, fortuitously, the distribution between sex groups was similar to the distribution between vaccine dose groups, and therefore the assumptions stated in the sample size calculation could be applied. Still, larger follow-up studies are necessary to further establish the role of each of these factors, including MPLA dosage.

Our study design considered the hypothesis that a fractional third dose could enhance SHM in memory B cells, which was based on an earlier study with the RTS,S/AS01_B_ malaria vaccine^26^. FxD recipients often had stronger serological responses, but differences could already be detected prior to the alteration in regimen and could therefore not be attributed to the fractional dose. These findings may stem from the relatively large proportion of female individuals in the FxD group (64%) compared to the FD group (54%), however differences between vaccine groups could also be detected between female participants, but not males (Supplementary Fig. 4b). In contrast to our initial hypothesis, fractional dose boosting did not appear to have a positive impact on SHM. It is therefore likely that the differences found for the RTS,S/AS01_B_ malaria vaccine – inspiring our study design – were caused by the extended time between vaccinations more so than the dose reduction^26^, demonstrating the need for sufficiently long vaccination intervals. Our finding that SHM increased substantially from week 26 to week 48 in the absence of additional vaccination reinforces the thought that time is an important factor contributing to SHM. This is in line with observations made in the COVID-19 field, showing that GCs can persist up to six months after infection or vaccination with increased SHM over time as a result^34,35^.

The MPLA-adjuvanted ConM SOSIP.v7 protein vaccine was previously tested in NHPs^12,13^. Given the differences in for example the naive BCR repertoire, we wondered how predictive NHPs were for humans. Similar to humans, NHPs developed potent autologous neutralisation, but no neutralisation breadth. Given the differences in analytical pathways (e.g., the use of serum vs. monoclonal antibodies; the pre-selection of V1V2 targeting Env-specific B cells in the single cell FACS sort in NHPs, but not humans), we were unable to directly compare antibody specificities. Nevertheless, while V1V2V3-targeting mAbs were found to be the main contributors to ConM-serum neutralisation in NHPs, in the human study this particular epitope appeared to be less dominant. Further epitope specification warrants follow-up analyses including the isolation and characterisation of human mAbs. As the NHP experiments included only female animals, differences between sexes could not be compared. Overall, we conclude that the NHP studies were predictive for humans, with some differences.

In conclusion, the MPLA-adjuvanted ConM SOSIP.v7 protein vaccine is safe and capable of eliciting cross-reactive binding antibody responses in humans, making it an interesting boosting immunogen candidate. Whether the differences we have found in immunological outcomes between females and males are caused by (a combination of) genetic, hormonal or environmental factors remains to be determined. Nevertheless, sex-based differences in response to vaccines and adjuvants need to be considered moving forward. This is particularly the case for HIV-1 vaccines, where young women and girls – accounting for 45% of new HIV-1 infections globally and a staggering 63% of new infections in sub-Saharan Africa in 2024 – are among the ones most in need of an effective vaccine (UNAIDS Factsheet 2025). Based on our findings they might benefit from the use of an adjuvant with a high dose of TLR4 agonist. Moving forward, it will be crucial to evaluate whether (sex)differences in immunogenicity actually lead to variations in vaccine efficacy through more extensive studies. As such, it is essential to include an adequate number of female individuals (both pre- and post-menopausal) in future research.

## Methods

### Study design, randomisation and vaccination

The ACTHIVE-001 study was a single centre, randomised, uncontrolled, open-label phase 1 clinical trial conducted at the Amsterdam University Medical Centres, location University of Amsterdam, the Netherlands. Participants were block-randomised using ALEA Clinical to one of two vaccine groups. ConM SOSIP.v7 gp140 (“MSIP528”) and MPLA liposomes were manufactured according to Good Manufacturing Practice (GMP) standards by Polymun Scientific GmbH (Klosterneuburg, Austria). Participants assigned to the full dose (FD) group received 100 μg ConM SOSIP.v7 vaccine at baseline (day zero), eight weeks (pre-specified range, seven to nine weeks) and 24 weeks (range, 23 to 25 weeks). Fractional dose (FxD) participants received the full dose vaccine at baseline and 8 weeks, followed by a fractional dose of 20 μg ConM SOSIP.v7 vaccine at 24 weeks. All vaccinations were adjuvanted with 500 μg MPLA liposomes. Vaccines were admixed ad hoc per each administration and were delivered as 0.65 mL intramuscular injections in the deltoid muscle of the same arm. Participants returned to the study site for safety follow-up visits one day, one week, two weeks and three weeks post vaccination. A check-up by telephone was conducted four weeks after each vaccination. Long-term follow-up visits took place at 48 and 72 weeks.

### Study participants

Participants were eligible for enrolment if they were between 18 and 50 years, had provided written informed consent, were generally healthy, not infected with HIV and at low risk for HIV acquisition, which included participants on a pre-exposure prophylaxis (PrEP) regimen. Participants were counselled to practice effective contraception until four months after the last vaccine administration. Exclusion criteria included, but were not limited to, history of drug sensitivity or allergy and receipt or planned receipt of (live attenuated) vaccines within 14 or 30 days of study vaccine administration (dependent on vaccine type). A full overview of in- and exclusion criteria is provided in the “Supplementary Methods”. Participants of childbearing capacity underwent urine testing for pregnancy at baseline and prior to each study vaccination. All participants were tested for HIV at screening, baseline (day zero), 24 weeks and 48 weeks. Both fourth generation HIV antibody/antigen tests and HIV RNA tests were conducted, in order to differentiate between infection and vaccine-induced seropositivity. HIV test counselling and risk reduction counselling were provided during pre-defined visits. Risk of HIV-infection was determined through a risk-assessment questionnaire based on the national PrEP guidelines, formulated by the Dutch Association of HIV-treating physicians.

### Study procedures

Peripheral-blood mononuclear cells (PBMCs) were isolated by density gradient centrifugation and cryopreserved in liquid nitrogen at each study visit. Serum and plasma were isolated at each study visit following and including baseline and archived at −80 °C. Leukapheresis procedures were performed prior to baseline and at two weeks following second vaccination. In order to obtain a minimum of 2.0 × 109 mononuclear cells, five liters of blood were processed at each time point, following local Donor Lymphocyte Infusion preparation protocol. Procedures were performed using the Spectra Optia Apheresis System. Vital functions were measured prior to, during and after the procedure. Safety bloods were collected prior to each procedure. Blood samples were collected in a 750 mL Leukapac collection bag containing Anticoagulant Citrate Dextrose (ACD) Solution A. Fine needle aspirates (FNA) of draining axillary lymph nodes were performed three weeks after the first and second vaccination. A detailed account of this procedure and outcomes will be described in a follow-up report.

Primary and secondary study endpoints have been listed below. This includes all safety endpoints and key immunogenicity endpoints such as Env-specific antibody binding and neutralisation. A full overview of primary, secondary and exploratory endpoints – indicating those included in this work - is presented in the “Supplementary Methods”.

### Safety endpoints

Primary safety endpoints included the proportion of participants with ≥ Grade 3 adverse events (AEs) during each seven-day post-vaccination period; the proportion of participants with ≥ Grade 3 and/or vaccine related AEs during each 28-day post-vaccination period and the proportion of participants with vaccine-related serious adverse events (SAE) throughout the entire study period (until close-out at 72 weeks). Causality in relationship to the vaccine was determined to be unrelated, unlikely, possibly, probably or definitely related according to pre-specified criteria. All AEs and SAEs were documented at each visit and were graded by the Table for Grading the Severity of Adult and Paediatric Adverse Events (corrected version 2.1 July 2017) as recommended by the Division of AIDS of the National Institute of Allergy and Infectious Diseases (NIAID).

### Binding antibody multiplex assay (BAMA) (Duke University)

Serum HIV-1 IgG responses against antigens (ConM SOSIP.v7-Biotin, ConM-BG505 V1V2 SOSIP.v7 Avi-Biotin, BG505 SOSIP.v8.1-GT1.1 Avi-Biotin, BG505 SOSIP.v8-GT1.1 Super-KO Avi-Biotin, BG505 SOSIP.v8.1-GT1.1 Apex-KO Avi-Biotin, BG505 SOSIP.v4.1-GT1.1 CD4bs-KO Avi-Biotin, BG505 SOSIP.v5 base KO + 613 T aviB, BG505 SOSIP.664 AviB, and the off-target gp41) were measured on a Bio-Plex instrument (Bio-Rad) using a standardised custom HIV-1 Luminex assay^49^. A cross-clade SOSIP trimer panel was also used to assess binding breadth - SDV TRO11 SOSIP.v9 Avi-His Stable, 25710 SOSIP.v4.2 Avi, CH119 SOSIP.v9 Avi, BJOX SOSIP.v4.2 Avi, 246-F3 SOSIP.v4.2 Avi – provided by the Moore lab at Weill Cornell, New York, NY. The readout is background-subtracted median fluorescence intensity (MFI), where background refers to the antigen-specific plate-level control (i.e., a blank well containing antigen-conjugated beads run on each plate). The positive controls were human immunodeficiency virus immune globulin (HIVIG), bNAbs PGT145 IgG mAb (quaternary V1V2 apex-specific IgG), PGT151 IgG mAb (gp120/gp140 interface trimer-specific IgG), VRC01 IgG mAb (CD4bs-specific IgG bNAb), 2G12 IgG mAb (Glycan-specific IgG mAb). Germline forms of the following CD4bs- and V2-apex-specific bNAbs were used as controls: CD4bs-specific mAbs germline VRC01, germline 12A12, and germline NIH45-46 and V2-apex-specific mAbs germline PG9, germline PG16, and germline CH01. In addition, the following IgG mAbs were utilised as controls to verify the expected binding pattern to the various SOSIP trimers: PGT125 (V3-specific bNAb), F105 (CD4bs-specific non-bNAb), 17B CL2 (CD4 inducible-specific non-bNAb), and 19B CL2 (V3-specific non-bNAb). 7B2 IgG mAb (gp41-specific) and RM19R and RM20A2 IgG mAb (base-specific) were used as controls for binding to off-target specificities. All mAbs except for RM19R and RM20A2 were provided by the Protein Production Facility at the Duke University Human Vaccine Institute, Durham NC. Blank beads and blank wells (antigen-coupled beads + detection Ab) served as negative controls for non-specific binding. If the blank bead negative control exceeded 5000 MFI, the sample was repeated. If the repeated value exceeded 5000 MFI, the sample was excluded from analysis due to high background. Samples are tested in replicates and a mean of two replicates was reported, after blank well and blank bead subtraction [net MFI, mean fluorescence intensity]. The area under the titration curve (AUTC) was calculated over the dilution series [1:50, 1:250, 1:1250, 1:6250, 1:31250, 1:156250] for the net MFI for each individual study participant by visit and antigen using the trapezoidal integration method with truncation at zero for negative net MFI values, and/or truncation at 22,000 for net MFI values greater than 22,000.

### IgG ELISA (Imperial College of Science, Technology and Medicine London)

Antigen specific IgG antibodies were measured in sera using in-house standardised conventional ELISA platforms^10,50^. In brief, 96-well medium-binding plates (Greiner, Kremsmünster, Austria) were coated with anti-human kappa and lambda light chain specific mouse antibodies (Southern Biotech, Birmingham, AL) at 1:1 ratio diluted 1:500 in PBS (Sigma-Aldrich, St. Louis, MO), or antigen (1 µg/mL ConM or ConS) for one hour at 37 °C. After blocking with block buffer (5% BSA (Sigma-Aldrich, St. Louis, MO), 0.05% Tween-20 (Fisher, Pittsburgh, PA) in D-PBS (Sigma-Aldrich)) samples were initially screened at 1:100 dilution (then titrated to optimal dilutions). Serial dilutions (1:5) of IgG standard (purified human IgG starting at 1 µg/mL (Sigma-Aldrich, St. Louis, MO) were added in triplicate to kappa/lambda capture Ab-coated wells and incubated for one hour at 37 °C. Secondary Ab, HRP-conjugated anti-human IgG (Sigma-Aldrich, St. Louis, MO), was added at 1:20,000 dilution, respectively, and incubated for one hour at 37 °C. Plates were developed with SureBlue TMB substrate (KPL, Insight Biotechnology, London, UK). The reaction was stopped after five minutes by adding TMB stop solution (KPL, Insight Biotechnology) and the absorbance read at 450 nm on a VersaMax 96 well microplate reader (Molecular Devices, Sunnyvale, CA). The ELISA data were expressed as positive if the blank-subtracted OD 450 nm was above the pre-determined cut-off of OD 0.2 nm and values were on the linear range of the curve. To ensure assay sensitivity, a positive control composed of positive pooled plasma samples was used. Analyses of the data were performed using SoftMax Pro GxP software (version 7, Molecular Devices, Sunnyvale, CA).

### Serum neutralisation assay (Duke University and Ospedale San Raffaele)

TZM-bl cell neutralisation assays using Env-pseudotyped viruses were performed at Duke University (DU)^51,52^ and Ospedale San Raffaele (OSR)^53^ as previously described. NAbs and sera were measured as a function of reductions in luciferase (Luc) reporter gene expression after a single round of infection in TZM-bl cells^51–53^. In short, at Duke University, a pre-titrated dose of virus was incubated with serial 3-fold dilutions of heat-inactivated (56 °C, 30 min) serum samples in duplicate in a total volume of 150 μl for one hour at 37 °C in 96-well flat-bottom culture plates. Freshly trypsinised cells ((10,000 cells in 100 μl (DU) or 75 μl (OSR) of growth medium (GM) containing 75 μg/mL (DU) or 45 μg/mL (OSR) DEAE dextran) were added to each well. One set of control wells received cells + virus (virus control) and another set received cells only (background control). At OSR, after 48 h of incubation, the medium was removed and 50 μl of Bright-Glo reagent (Promega, Madison, Wisconsin, USA) diluted 1:2 with GM was dispensed into each well. The plate was incubated at room temperature for two minutes to allow complete cell lysis. 40 μl was transferred to a corresponding 96-well white plate and analysed in a luminometer (Mithras (Berthold, Germany)). At DU, after 48 h of incubation, 100 µl of cells was transferred to a 96-well black solid plate (Costar) for measurements of luminescence using the Britelite Luminescence Reporter Gene Assay System (PerkinElmer Life Sciences). Neutralisation titres are the dilution (serum/plasma samples) or concentration (mAbs) at which relative luminescence units (RLU) were reduced by 50% or 80% compared to virus control wells after subtraction of background RLUs. Assay stocks of molecularly cloned Env-pseudotyped viruses were prepared by transfection in 293 T/17 cells (American Type Culture Collection) and titrated in TZM-bl cells as described^51,52^. All samples were tested against the autologous ConM virus and only week 26 samples were tested against a panel representative of global HIV-1 varieties (Ce1176_A3, TRO.11, 25710-2.43, BJOX002000.03.2, CH119.10, X1632_S2_B10, 246-F3_C10_2, Ce703010217_B6, CNE55^54^). This assay has been formally optimised and validated^55^ and was performed in compliance with Good Clinical Laboratory Practices, including participation in a formal proficiency testing programme^56^. Additional information on the assay and all supporting protocols may be found at: http://www.hiv.lanl.gov/content/nab-reference-strains/html/home.htm. All ConM SOSIP.v7 depletion experiments were performed at the Amsterdam UMC. Reagents contain the D368R mutation to abrogate binding to CD4 on the TZM-bl receptor cell line, as described previously^9^.

### Luminex IgG1-4 subtype assay

Serum HIV-1 IgG 1 (IgG1), 2 (IgG2), 3 (IgG3) and 4 (IgG4) subtype responses specific for ConM SOSIP.v7 were measured using a custom Luminex assay as previously described for other antigens^57^. ConM SOSIP.v7 was conjugated to Magplex microspheres (Luminex) using a two-step carbodiimide reaction. Microspheres were washed with 100 mM monobasic sodium phosphate pH 6.2 and then activated with Sulfo-N-Hydroxysulfosuccinimide (Thermo Fisher Scientific) and 1-Ethyl-3-(3-dimethylaminopropyl) carbodiimide (Thermo Fisher Scientific) for 30 minutes on a rotator at room temperature. The microspheres were washed twice with 50 mM MES pH 5.0, then ConM SOSIP.v7 was added at a ratio of 75 µg per 12.5 million microspheres and this was incubated for three hours on a rotator at room temperature. The microspheres were blocked for 30 min with blocking buffer (PBS containing 2% BSA, 3% foetal calf serum and 0.02% Tween-20 at pH 7.0) and finally stored at 4 °C in PBS containing 0.05% sodium azide until use. BSA-blocked microspheres with no protein were included as a negative control. To assess ConM SOSIP.v7-specific IgG1-4 responses, sera were diluted 1:50,000 for IgG1, 1:100 for IgG2 and IgG4, and 1:500 for IgG3 (based on prior optimisation experiments) in blocking buffer. 50 µl of diluted sera was incubated with 750 of each ConM SOSIP.v7-conjugated beads and negative control beads in 50 µl blocking buffer overnight on a shaker at 4 °C. The next day, plates were washed with PBS + 0.05% Tween-20 and then incubated with 50 µl mouse anti-human IgG1-PE, mouse anti-human IgG2-PE, mouse anti-human IgG3-PE or mouse anti-human IgG4-PE (Southern Biotech) at 1.3 µg/mL in blocking buffer. Finally, plates were washed with PBS + 0.05% Tween-20 and the microspheres were resuspended in 70 µl MAGPIX drive fluid (Luminex) and read-out on the MAGPIX instrument (Luminex). Resulting Median Fluorescence Intensity values were subtracted with MFI values from microsphere and buffer only wells.

### IgDiscover

Individualised germline immunoglobulin heavy chain variable (IGHV) gene databases from all study participants were produced from leukapheresis samples obtained before immunisation. In brief, IgM libraries for deep sequencing of antibody repertoires were generated with 5’ multiplex PCR from total PBMC mRNA, as previously described^58^. The output library was analysed with IgDiscover to infer the germline gene IGHV alleles^33^. The IGHV database utilised as an input database for the IgDiscover analysis was obtained from genomic DNA sequencing^58^.

### Electron microscopy-based polyclonal epitope mapping (EMPEM)

Antibody specificities elicited by the adjuvanted ConM SOSIP.v7 vaccine were studied by electron microscopy-based polyclonal epitope mapping (EMPEM), whereby total serum IgG is digested into antigen binding fragments (Fab), complexed with Env (SOSIP) trimers and imaged using negative-stain electron microscopy (NS-EM)^27^. IgG isolation from serum samples was performed using two different methods. All serum samples were initially heat-inactivated at 56 °C for one hour. For samples cvd890_516 − 231, 0.5 mL of serum was added to an equal volume of Protein G resin and allowed to incubate for 96 hours on a rolling platform at 4 °C. The resin was washed with PBS three times before IgG elution. Eluted samples were buffer exchanged into PBS. For samples cvd890_994 − 790, 0.5 mL of each serum was filtered using a 0.2 µm syringe filter unit. Samples were diluted in PBS up to 2 mL. Tween-20 detergent was added to each sample for a final concentration of 1% (v/v) and mixed until dissolved. Each sample was incubated at room temperature for 30 minutes before transferring to a 96 deep-well plate. An ALIAS autosampler was used to inject samples onto an in-house packed 6 mL CaptureSelect IgG-Fc column (Thermo Fisher Scientific) on an AKTAPure system (Cytiva). Fractions corresponding to the IgG peak were collected and buffer-exchanged into PBS. IgG was digested into Fab using papain (Sigma Aldrich) and buffer exchanged into TBS. The resulting “dirty Fabs” containing papain, undigested IgG, and Fc were complexed with 15 µg total of a 50%/50% mixture of ConM SOSIP.v7 and ConM SOSIP.v9 and 0.5–1.0 mg of polyclonal Fab mixture. Complexes were incubated overnight at room temperature and purified over a Superdex 200 Increase column on an AKTAPure system (Cytiva). Fractions corresponding to the complex peak were collected and deposited onto glow-discharged copper mesh grids at a concentration of ~0.03 mg/ml. Grids were stained with 2% uranyl formate for 90 s. Automated data collection was set up using Leginon^59^ on a 120 keV FEI Tecnai Spirit with a Tietz 4 K x 4 K camera. Micrographs were saved in the Appion database^60^ and scanned for particles using DoGpicker^61^. Particles were processed in Relion 3.0^62^ and 3D models were segmented with UCSF Chimera^63^.

### SHM and IGHV gene analysis by single cell RNAseq

PBMCs samples at week 26 and week 48 were used to determine IGHV gene usage and SHM levels for all per-protocol participants (*N* = 23). 10X single cell RNAseq (scRNAseq) was used to determine B-cell receptor sequences (BCR) at the single cell level. To combine all 46 samples in one 10x scRNAseq run, samples were divided over four lanes and TotalSeq C anti-human hashtag antibodies (BioLegend), targeting CD298 andβ2 microglobulin, were used to multiplex up to 12 samples. To ensure lane variability was minimal, samples were randomised across lanes, with the two time points per participant separated in different lanes. Cells were thawed in RPMI 1640 medium supplemented with 20% Foetal Calf Serum (FCS). Next, CD3+ cells were depleted using the plate-based Easy-sep Human CD3 positive selection kit by Stem Cell Technologies. Approximately 5% of every sample was taken aside and mixed together per lane to sort unspecific cells. Cells were stained with TruStain FcX (BioLegend) and TotalSeq C hashtag antibodies according to the manufacturer’s instructions. Subsequently, cells were stained with ConM SOSIP.v7 loaded on (dCode) Klickmer (Immudex) containing a single stranded DNA oligonucleotide barcode and fluorochrome for detection by FACS and scRNAseq analysis. ConM SOSIP.v7 was incubated with PE dCode Klickmer or APC Klickmer at a 5:1 protein to klickmer ratio for 1 h. Next, 100 uM Biotin was added to bind any remaining available spots on the klickmer dextramer. In addition, cells were simultaneously stained with dCode klickmer loaded with Biotin only to remove any klickmer specific background. Finally, cells were incubated with a FACS phenotype panel consisting of CD4 (OKT4) APC- eF780 (Thermo Fisher), CD14 (M5E2) APC- eF780 (Thermo Fisher) CD16 (ebioCD16) APC- eF780 (eBioScience), CD19 (HIB19) AF700 (BioLegend), IgD (IA6-2) BV785 (BioLegend), CD3 (UCHT1) eF506 (Thermo Fisher) CD27 (M-T271) BV421 (BioLegend). CD20+ ConM SOSIP.v7 specific B cells were bulk sorted using a BD FacsAria II sorter. Due to low cell counts, unspecific cells were sorted from all samples per lane to fill up the lanes to approximately 10,000 cells. Transcriptomics, feature barcode, and VDJ libraries were developed per lane using the Chromium Next GEM Single Cell 5’ Kit, 5’ Feature Barcode Kit, and Chromium Single Cell Human BCR Amplification Kit (10X Genomics). Libraries were sequenced by Illumina sequencing and subsequently processed using the count pipeline in Cell Ranger. Next, filtered count matrices were further analysed using the Seurat scRNAseq analysis tool in the R programming language^64^. The HTODemux function of the Seurat package was used to demultiplex samples per lane. To further validate demultiplexing results, ConM SOSIP.v7 and biotin decoy Klickmer barcodes were used to only select ConM+ decoy- cells. Next, cells with high percentages of mitochondrial genes ( > 7.5%) and low number of housekeeping genes ( < 55 genes) were removed from the dataset as they were considered poor quality. Cells were normalised using centred log ratio (CLR) and scaled by Seurat function ScaleData. Data was dimensionally reduced using the RunPCA and RunUMAP functions in the Seurat package. The top 7–9 principal components (lane 1: 8, lane 2: 7, lane 3: 9, lane 4: 8) were used for Uniform Manifold Approximation and Projection (UMAP) analysis. FindNeighbours followed by FindClusters was used to cluster dimensionally reduced data based on a shared nearest neighbour modularity optimisation-based algorithm. Clusters were automatically annotated using the SingleR package and the built-in Monaco database^65^ (GEO Accession viewer (nih.gov)). To analyse SHM levels and IGHV gene usage in ConM SOSIP.v7 specific memory B-cells, memory B-cells were selected based on SingleR annotation and SHM levels. Cells with a memory B-cell annotation or a IGHV gene SHM level bigger than 0% were defined as memory B-cells. ggplot was used to visualise IGHV SHM levels and V gene usage.

### IL-6 determination via ELISA

IL-6 levels were determined in the supernatant of PBMCs following 24 h of stimulation. PBMCs (200.000/condition) were cultured in 200 µL RPMI medium enriched with 10% FCS (Biological Industries), 10 IU/mL penicillin (Thermo Fisher), 10 mg/mL streptomycin (Thermo Fisher), 2 mM L-glutamine (Lonza) and 10 IU/mL IL-2 (Invivogen) overnight prior to stimulation. Subsequently, PBMCs were stimulated in biological triplicates with ConM SOSIP.v7 glycoprotein (5 µg/mL), MPLA (30 µg/mL), ConM SOSIP.v7 plus MPLA (5 µg/mL; 30 µg/mL), LPS (10 ng/mL), GS9620 (10 µM), GS9688 (1 µM), CPG-ODN (5 µg/mL). After 24 hours of stimulation, supernatant was harvested and stored at −20 °C prior to analysis. Secretion of IL-6 proteins were measured by ELISA as described by the manufacturer (eBiosciences). OD450nm values were measured using BioTek synergy HT.

### TLR4 expression determination by flowcytometry and qPCR

PBMC were defrosted in IMDM containing 20% NCS and penicillin/streptomycin (all from Thermo Fisher Scientific, Waltham MA, USA) and counted on a Coulter Counter (Beckman Coulter, Fullerton CA, USA). 2 × 2.10^6^ cells were stained for flowcytometry and 2.10^6^ cells were lysed for RNA isolation, reverse transcription and qPCR. Flowcytometry staining was performed using the following antibodies: CD14 PE-Cy7 FITC (Thermo Fisher Scientific, Waltham MA, USA), CD16 APC (BioLegend, San Diego CA, USA), and TLR4 BV421 (BioLegend, San Diego CA, USA). Cells were fixed in FluoroFix (BioLegend, San Diego CA, USA) and measured on a FACSCanto II Flowcytometer (BD Biosciences, Franklin Lake NJ, USA). Data was analysed using FlowJo software (BD, Franklin Lake NJ, USA).

RNA was extracted from the 2.10^6^ PBMC using the RNeasy Plus kit (QIAGEN, Hilden, Germany) according to manufacturer’s protocol, followed by reverse transcription. RT setup comprised M-MLV Reverse Transcriptase (Promega, Madison WI, USA), RNAsin RNAse inhibitor (Promega, Madison WI, USA), dNTPs (Promega, Madison WI, USA), oligo dT primers and 10 ul of isolated RNA according to manufacturer’s protocol in a final volume of 20 ul. qPCR reactions for TLR4 and GAPDH as housekeeping gene were set up using FAST SYBR Green 2x Master Mix (Thermo Fisher Scientific, Waltham MA, USA) and the following primers: TLR4-F (5’ → 3’ CAACCTCCCCTTCTCAACCA), TLR4-R (5’ → 3’ GGGCTAAACTCTGGATGGGG), and GAPDH-F (5’ → 3’ GGCATGGACTGTGGTCATGA), GAPDH-R (5’ → 3’ TGCACCACCAACTGCTTAGC). 2 ul of 5x diluted cDNA was used as template in a final volume of 10 ul, in a 384 wells plate format. qPCR was performed on a QuantStudio 5 device (Thermo Fisher Scientific, Waltham MA, USA) using the following programme: a primary step of 95 °C 20 s; 40 cycles of 95 °C 1 s and 60 °C 20 s; a final melting curve determination step of 95 °C 1 s, 60 °C 20 s and a dissociation step to 95 °C with a ramp rate of 0.075 °C/s. qPCR data was analysed using QuantStudio Design & Analysis software (Thermo Fisher Scientific, Waltham MA, USA).

### Sample size and statistical analyses

The study sample size was selected based on comparable phase 1 HIV vaccine trials and was primarily powered to detect differences in adverse event rates and serum neutralisation titres between vaccine groups. Additionally, randomisation was stratified in order to facilitate the detection of potential sex-differences in study endpoints. Safety endpoints were evaluated on a modified intention-to-treat basis and included all participants who received at least one vaccination. Descriptive summary data (numbers and percentages) for all participants with any adverse event are presented for either the full cohort, per vaccine group or per sex at birth. Immunogenicity endpoints were measured for the per-protocol cohort, consisting of participants who received all three vaccinations and had completed the week 48 follow-up. The Mann–Whitney U test was used for independent sample comparisons and a Friedman test followed by a Dunn’s multiple comparison test was used for comparing three time points for the same individuals using GraphPad Prism version 9.3.1 (GraphPad Software, La Jolla, CA). All tests were two-tailed. A Bonferroni correction for multiple testing was applied to the sex-hormone correlation analyses, and a repeated measures two-way NOVA followed by a Tukey multiple comparisons test was applied to the PBMC stimulation assays. For the RNA-sequencing analyses, differences between groups and sex were calculated by Wilcoxon signed rank test, paired comparisons were calculated by donor median SHM paired Wilcoxon signed rank test.

### Inclusion and ethics

This study received approval from the Central Committee on Research Involving Human Subjects, the Ministry of Health, Welfare and Sports of the Netherlands and the Medical Research Ethics Committee of the Amsterdam University Medical Centres (previously ‘Academic Medical Centre’). All participants provided written informed consent. The clinical trial is registered at ClinicalTrials.gov under identification number NCT03961438.

### Reporting summary

Further information on research design is available in the Nature Portfolio Reporting Summary linked to this article.

## Supplementary information

Supplementary Information

Reporting Summary

Transparent Peer Review file

## Source data

Source Data

## Data Availability

The clinical raw data generated in this study are protected and are not available due to data privacy laws. The processed and coded antibody binding (IgG and IgG subtypes), neutralisation, sex hormone, IgDiscover, IL-6 and TLR 4 expression data generated in this study are provided in the Source Data file and through Figshare (10.6084/m9.figshare.29957945). A representative EMPEM reconstruction has been deposited to the Electron Microscopy Data Bank (EMDB) with accession code EMD-47765. The sequence data generated in this study are available in the European Nucleotide Archive under project code PRJEB96296. Any additional information is available from the corresponding authors upon request. Source data are provided with this paper.

## References

[1] Sharp, P. M. & Hahn, B. H. Origins of HIV and the AIDS pandemic. *Cold Spring Harb. Perspect Med.* **1**, a006841 (2011). 10.1101/cshperspect.a006841 PMC3234451 22229120

[2] Hemelaar, J. et al. Global and regional molecular epidemiology of HIV-1, 1990–2015: a systematic review, global survey, and trend analysis. *Lancet Infect. Dis*. 10.1016/S1473-3099(18)30647-9 (2019). 10.1016/S1473-3099(18)30647-9 30509777

[3] Gaudinski, M. R. et al. Safety and pharmacokinetics of the Fc-modified HIV-1 human monoclonal antibody VRC01LS: a Phase 1 open-label clinical trial in healthy adults. *PLoS Med.*10.1371/journal.pmed.1002493 (2018). 10.1371/journal.pmed.1002493 PMC5783347 29364886

[4] Corey, L. et al. Two randomized trials of neutralizing antibodies to prevent HIV-1 acquisition. *N. Engl. J. Med.* **384**, 1003–1014 (2021). 33730454 10.1056/NEJMoa2031738 PMC8189692

[5] Shingai, M. et al. Passive transfer of modest titers of potent and broadly neutralizing anti-HIV monoclonal antibodies block SHIV infection in macaques. *J. Exp. Med.* **211**, 2061–2074 (2014). 25155019 10.1084/jem.20132494 PMC4172223

[6] Caskey, M. Broadly neutralizing antibodies for the treatment and prevention of HIV infection. *Curr. Opin. HIV AIDS* **15**, 329–346 (2020). 10.1097/COH.0000000000000600 PMC7340121 31764199

[7] Sanders, R. W. & Moore, J. P. Progress on priming HIV-1 immunity. *Science (1979)* **384**, 738–739 (2024). 10.1126/science.adp3459 38753801

[8] Sanders, R. W. et al. A next-generation cleaved, soluble HIV-1 Env trimer, BG505 SOSIP.664 gp140, expresses multiple epitopes for broadly neutralizing but not non-neutralizing antibodies. *PLoS Pathog.* **9**, e1003618 (2013). 24068931 10.1371/journal.ppat.1003618 PMC3777863

[9] Sliepen, K. et al. Structure and immunogenicity of a stabilized HIV-1 envelope trimer based on a group-M consensus sequence. *Nat. Commun.* **10**, 2355 (2019). 31142746 10.1038/s41467-019-10262-5 PMC6541627

[10] Reiss, E. I. M. M. et al. Fine-mapping the immunodominant antibody epitopes on consensus sequence-based HIV-1 envelope trimer vaccine candidates. *NPJ Vaccines* **7**, 152 (2022). 36433972 10.1038/s41541-022-00576-9 PMC9700725

[11] Pollock, K. M. et al. Experimental medicine study with stabilised native-like HIV-1 Env immunogens drives long-term antibody responses, but lacks neutralising breadth. *EBioMedicine* **112**, 105544 (2025). 10.1016/j.ebiom.2024.105544 PMC11753977 39753033

[12] Van Tilbeurgh, M. et al. Innate cell markers that predict anti-HIV neutralizing antibody titers in vaccinated macaques. *Cell Rep. Med.* **3**, 100751 (2022). 10.1016/j.xcrm.2022.100751 PMC9588994 36167072

[13] Sliepen, K. et al. Interplay of diverse adjuvants and nanoparticle presentation of native-like HIV-1 envelope trimers. *NPJ Vaccines* **6**, 103 (2021). 34404812 10.1038/s41541-021-00364-x PMC8371121

[14] Roman, F. et al. Adjuvant system AS01: from mode of action to effective vaccines. *Expert Rev. Vaccines* **23**, 715–729 (2024). 39042099 10.1080/14760584.2024.2382725

[15] Alving, C. R., Peachman, K. K., Matyas, G. R., Rao, M. & Beck, Z. Army Liposome Formulation (ALF) family of vaccine adjuvants. *Expert Rev. Vaccines* **19**, 279–292 (2020). 32228108 10.1080/14760584.2020.1745636 PMC7412170

[16] Klein, S. L. & Flanagan, K. L. Sex differences in immune responses. *Nat. Rev. Immunol.* **16**, 626–638 (2016). 27546235 10.1038/nri.2016.90

[17] Klein, S. L., Jedlicka, A. & Pekosz, A. The Xs and Y of immune responses to viral vaccines. *Lancet Infect Dis* **10**, 338–349 (2010). 10.1016/S1473-3099(10)70049-9 PMC6467501 20417416

[18] Lakshmikanth, T. et al. Immune system adaptation during gender-affirming testosterone treatment. *Nature* **633**, 155–164 (2024). 39232147 10.1038/s41586-024-07789-z PMC11374716

[19] Giefing-Kröll, C., Berger, P., Lepperdinger, G. & Grubeck-Loebenstein, B. How sex and age affect immune responses, susceptibility to infections, and response to vaccination. *Aging Cell* **14**, 309–321 (2015). 10.1111/acel.12326 PMC4406660 25720438

[20] Potluri, T. et al. Age-associated changes in the impact of sex steroids on influenza vaccine responses in males and females. *NPJ Vaccines* **4**, 29 (2019). 31312529 10.1038/s41541-019-0124-6 PMC6626024

[21] Tadount, F. et al. Sex differences in the immunogenicity and efficacy of seasonal influenza vaccines: a meta-analysis of randomized-controlled trials. *Open Forum Infect. Dis.* **11**, ofae222 (2024). 10.1093/ofid/ofae222 PMC11088355 38737434

[22] Fink, A. L. & Klein, S. L. Sex and gender impact immune responses to vaccines among the elderly. *Physiology* **30**, 408–416 (2015). 26525340 10.1152/physiol.00035.2015 PMC4630198

[23] Anticoli, S. et al. Sex differences in response to HBV vaccination in a cohort of health care workers. *Vaccin. X* **22**, 100605 (2025). 10.1016/j.jvacx.2024.100605 PMC11731748 39811673

[24] Bachmann, M. et al. Disparities in response to mRNA SARS-CoV-2 vaccines according to sex and age: a systematic review. *N. Microbes N. Infect.* **63**, 101551 (2025). 10.1016/j.nmni.2024.101551 PMC11726804 39807161

[25] Haynes, B. F., Kelsoe, G., Harrison, S. C. & Kepler, T. B. B-cell-lineage immunogen design in vaccine development with HIV-1 as a case study. *Nat Biotechnol* **30**, 423–433 (2012). 10.1038/nbt.2197 PMC3512202 22565972

[26] Regules, J. et al. Fractional third and fourth dose of RTS,S/AS01 malaria candidate vaccine: a phase 2a controlled human malaria infection and immunogenicity study. *J. Infect. Dis.* **214**, jiw237 (2016). 10.1093/infdis/jiw237 27296848

[27] Turner, H. et al. Protocol for analyzing antibody responses to glycoprotein antigens using electron-microscopy-based polyclonal epitope mapping. *STAR Protoc.* **4**, 102476 (2023). 37516970 10.1016/j.xpro.2023.102476 PMC10400963

[28] Hahn, W. O. et al. Use of 3M-052-AF with Alum adjuvant in HIV trimer vaccine induces human autologous neutralizing antibodies. *J. Exp. Med.* **221**, e20240604 (2024). 39235529 10.1084/jem.20240604 PMC11380150

[29] Turner, H. L. et al. Disassembly of HIV envelope glycoprotein trimer immunogens is driven by antibodies elicited via immunization. *Sci. Adv.* **7**, eabh2791 (2021). 10.1126/sciadv.abh2791 PMC8318364 34321200

[30] Olia, A. S. et al. Soluble prefusion-closed HIV-envelope trimers with glycan-covered bases. *iScience* **26**, 107403 (2023). 37554450 10.1016/j.isci.2023.107403 PMC10404741

[31] Lee, J. H. et al. Antibodies to a conformational epitope on gp41 neutralize HIV-1 by destabilizing the Env spike. *Nat. Commun.* **6**, 8167 (2015). 26404402 10.1038/ncomms9167 PMC4586043

[32] Medina-Ramírez, M. et al. Design and crystal structure of a native-like HIV-1 envelope trimer that engages multiple broadly neutralizing antibody precursors in vivo. *J. Exp. Med.*10.1084/jem.20161160 (2017). 10.1084/jem.20161160 PMC5584115 28847869

[33] Corcoran, M. M. et al. Production of individualized V gene databases reveals high levels of immunoglobulin genetic diversity. *Nat. Commun. 2016* **7**, 1–14 (2016). 10.1038/ncomms13642 PMC5187446 27995928

[34] Laidlaw, B. J. & Ellebedy, A. H. The germinal centre B cell response to SARS-CoV-2. *Nat. Rev. Immunol.* **22**, 7–18 (2022). 34873279 10.1038/s41577-021-00657-1 PMC8647067

[35] Kim, W. et al. Germinal centre-driven maturation of B cell response to mRNA vaccination. *Nature* **604**, 1–8 (2022). 10.1038/s41586-022-04527-1 PMC9204750 35168246

[36] Joyce, C., Burton, D. R. & Briney, B. Comparisons of the antibody repertoires of a humanized rodent and humans by high throughput sequencing. *Sci. Rep.* **10**, 1120 (2020). 31980672 10.1038/s41598-020-57764-7 PMC6981180

[37] Jego, G., Bataille, R. & Pellat-Deceunynck, C. Interleukin-6 is a growth factor for nonmalignant human plasmablasts. *Blood* **97**, 1817–1822 (2001). 11238125 10.1182/blood.v97.6.1817

[38] Arkatkar, T. et al. B cell–derived IL-6 initiates spontaneous germinal center formation during systemic autoimmunity. *J. Exp. Med.* **214**, jem.20170580 (2017). 10.1084/jem.20170580 PMC5679179 28899868

[39] Fensterheim, B. et al. The TLR4 agonist monophosphoryl lipid A drives broad resistance to infection via dynamic reprogramming of macrophage metabolism. *J. Immunol.* **200**, ji1800085 (2018). 10.4049/jimmunol.1800085 PMC5964009 29686054

[40] Owen, A. et al. MyD88-dependent signaling drives toll-like receptor-induced trained immunity in macrophages. *Front Immunol.* **13**, 1044662 (2022). 36439136 10.3389/fimmu.2022.1044662 PMC9692127

[41] Romero, C. et al. The Toll-like receptor 4 agonist monophosphoryl lipid A augments innate host resistance to systemic bacterial infection. *Infect. Immun.* **79**, 3576–3587 (2011). 21646453 10.1128/IAI.00022-11 PMC3165493

[42] Cohen, K. et al. A first-in-human germline-targeting HIV nanoparticle vaccine induced broad and publicly targeted helper T cell responses. *Sci. Transl. Med.* **15**, eadf3309 (2023). 37224227 10.1126/scitranslmed.adf3309 PMC11036875

[43] Alving, C. R., Rao, M., Steers, N. J., Matyas, G. R. & Mayorov, A. V. Liposomes containing lipid A: an effective, safe, generic adjuvant system for synthetic vaccines. *Expert Rev. Vaccines* **11**, 733–744 (2012). 22873129 10.1586/erv.12.35

[44] Hoebe, C. J. P. A., Vermeiren, A. P. A. & Dukers-Muijrers, N. H. T. M. Revaccination with Fendrix® or HBVaxPro® results in better response rates than does revaccination with three doses of Engerix-B® in previous non-responders. *Vaccine* **30**, 6734–6737 (2012). 22981848 10.1016/j.vaccine.2012.08.074

[45] Lehtinen, M. et al. Overall efficacy of HPV-16/18 AS04-adjuvanted vaccine against grade 3 or greater cervical intraepithelial neoplasia: 4-year end-of-study analysis of the randomised, double-blind PATRICIA trial. *Lancet Oncol.* **13**, 89–99 (2012). 22075171 10.1016/S1470-2045(11)70286-8

[46] Grobben, M. et al. Induction of HIV-1-specific antibody-mediated effector functions by native-like envelope trimers in humans. *PLoS Pathog.* **21**, e1013614 (2025). 41118401 10.1371/journal.ppat.1013614 PMC12551954

[47] Guéry, J.-C. Sex differences in primary hiv infection: revisiting the role of TLR7-driven type 1 IFN production by plasmacytoid dendritic cells in women. *Front Immunol.* **12**, 729233 (2021). 34512664 10.3389/fimmu.2021.729233 PMC8432934

[48] Qiao, Q. et al. AID recognizes structured DNA for class switch recombination. *Mol. Cell* **67**, 361–373.e4 (2017). 28757211 10.1016/j.molcel.2017.06.034 PMC5771415

[49] D, T. G. et al. Initial B-cell responses to transmitted human immunodeficiency virus type 1: virion-binding immunoglobulin M (IgM) and IgG antibodies followed by plasma anti-gp41 antibodies with ineffective control of initial viremia. *J. Virol.* **82**, 12449–12463 (2008). 18842730 10.1128/JVI.01708-08 PMC2593361

[50] Cheeseman, H. M. et al. Combined skin and muscle DNA priming provides enhanced humoral responses to a human immunodeficency virus type 1 clade C envelope vaccine. *Hum. Gene Ther.*10.1089/hum.2018.075 (2018). 10.1089/hum.2018.075 PMC6214652 30027768

[51] Li, M. et al. Human immunodeficiency virus type 1 env clones from acute and early subtype B infections for standardized assessments of vaccine-elicited neutralizing antibodies. *J. Virol.*10.1128/jvi.79.16.10108-10125.2005 (2005). 10.1128/JVI.79.16.10108-10125.2005 PMC1182643 16051804

[52] Montefiori, D. C. Measuring HIV neutralization in a luciferase reporter gene assay. *Methods Mol. Biol.* **485**, 395–405 (2009). 19020839 10.1007/978-1-59745-170-3_26

[53] Heyndrickx, L. et al. International network for comparison of HIV neutralization assays: The neutnet report II. *PLoS One*10.1371/journal.pone.0036438 (2012). 10.1371/journal.pone.0036438 PMC3348930 22590544

[54] De Camp, A. et al. Global panel of HIV-1 Env reference strains for standardized assessments of vaccine-elicited neutralizing antibodies. *J. Virol.* **88**, 2489–2507 (2014). 24352443 10.1128/JVI.02853-13 PMC3958090

[55] Sarzotti-Kelsoe, M. et al. Optimization and validation of the TZM-bl assay for standardized assessments of neutralizing antibodies against HIV-1. *J. Immunol. Methods* **409**, 131–146 (2014). 24291345 10.1016/j.jim.2013.11.022 PMC4040342

[56] Todd, C. A. et al. Development and implementation of an international proficiency testing program for a neutralizing antibody assay for HIV-1 in TZM-bl cells. *J. Immunol. Methods* **375**, 57 (2012). 21968254 10.1016/j.jim.2011.09.007 PMC3332116

[57] Grobben, M. et al. Cross-reactive antibodies after SARS-CoV-2 infection and vaccination. *Elife* **10**, e70330 (2021). 34812143 10.7554/eLife.70330 PMC8610423

[58] Vázquez Bernat, N. et al. High-quality library preparation for NGS-based immunoglobulin germline gene inference and repertoire expression analysis. *Front. Immunol.* **10**, 660 (2019). 10.3389/fimmu.2019.00660 PMC6459949 31024532

[59] Suloway, C. et al. Automated molecular microscopy: the new Leginon system. *J. Struct. Biol.* **151**, 41–60 (2005). 10.1016/j.jsb.2005.03.010 15890530

[60] Lander, G. C. et al. Appion: an integrated, database-driven pipeline to facilitate EM image processing. *J. Struct. Biol.* **166**, 95–102 (2009). 10.1016/j.jsb.2009.01.002 PMC2775544 19263523

[61] Voss, N. R., Yoshioka, C. K., Radermacher, M., Potter, C. S. & Carragher, B. DoG Picker and TiltPicker: software tools to facilitate particle selection in single particle electron microscopy. *J. Struct. Biol.* **166**, 205–213 (2009). 19374019 10.1016/j.jsb.2009.01.004 PMC2768396

[62] Zivanov, J. et al. New tools for automated high-resolution cryo-EM structure determination in RELION-3. *Elife* **7**, e42166 (2018). 10.7554/eLife.42166 PMC6250425 30412051

[63] Pettersen, E. F. et al. UCSF Chimera - A visualization system for exploratory research and analysis. *J. Comput. Chem.* **25**, 1605–1612 (2004). 10.1002/jcc.20084 15264254

[64] Hao, Y. et al. Integrated analysis of multimodal single-cell data. *Cell* **184**, 3573–3587.e29 (2021). 34062119 10.1016/j.cell.2021.04.048 PMC8238499

[65] Aran, D. et al. Reference-based analysis of lung single-cell sequencing reveals a transitional profibrotic macrophage. *Nat. Immunol.* **20**, 163–172 (2019). 30643263 10.1038/s41590-018-0276-y PMC6340744

