# Supplementary material for: HIV-1 envelope trimer vaccine induces sex-associated differences in antibody responses: a phase 1 clinical trial
Source: Nat Commun. 2025 Nov 21;16:10250. doi: 10.1038/s41467-025-65101-7 (PMC12639139; doi:10.1038/s41467-025-65101-7)
Supplement: Supplementary file 1 — Supplementary Information [file 41467_2025_65101_MOESM1_ESM.pdf]

HIV-1 envelope trimer vaccine induces sex-associated differences in antibody responses: a phase 1 clinical trial

SUPPLEMENTARY INFORMATION

Supplementary Figures

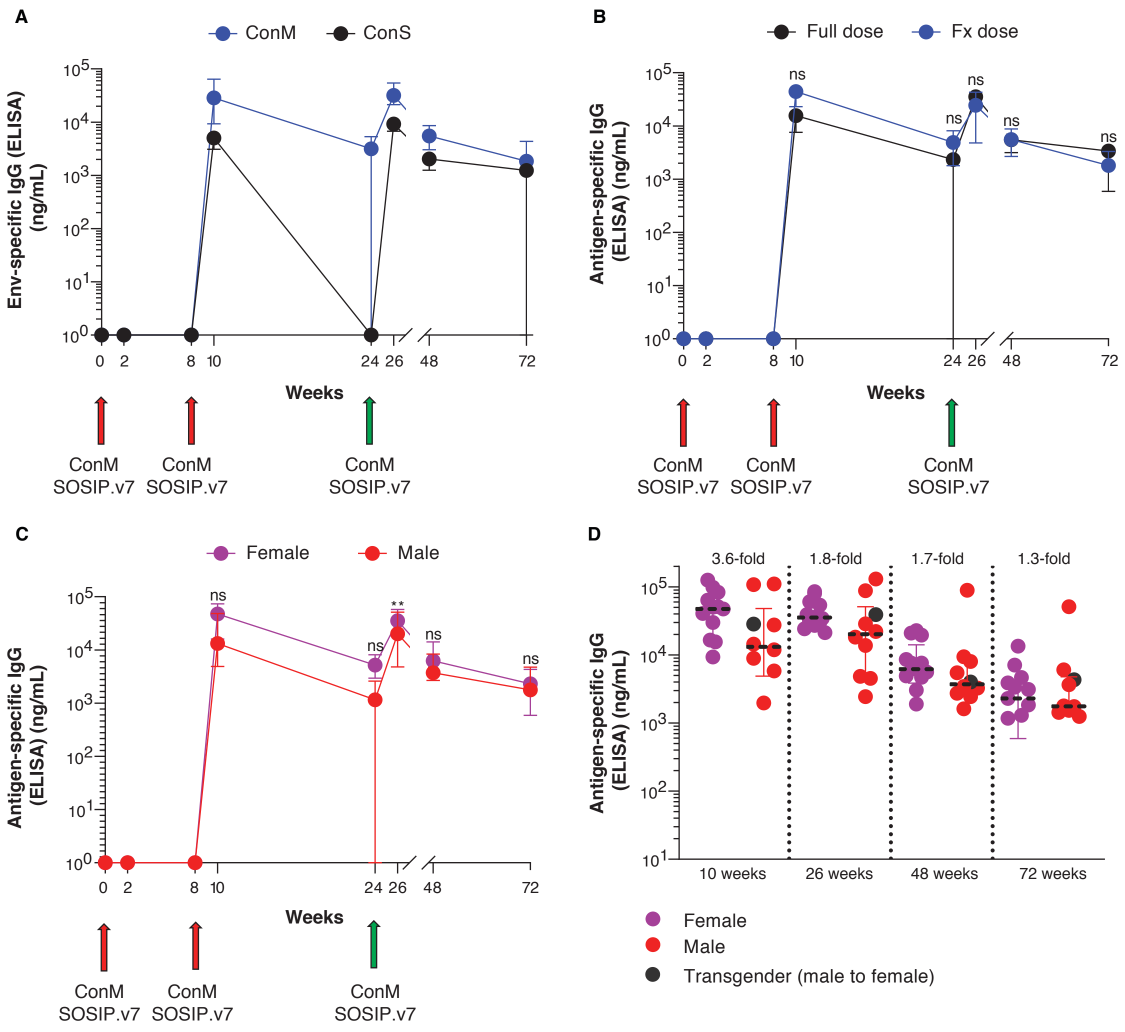

Supplementary Fig. 1. ConM SOSIP.v7-specific antibody binding (ELISA). **a.** ConM SOSIP.v7-specific and ConS SOSIP.v9-specific IgG levels measured by enzyme-linked immuno sorbent assay (ELISA). Values indicate median IgG levels (ng/mL) and are depicted at each vaccination baseline (0, 8 and 24 weeks), two weeks post-vaccination (two, 10 and 26 weeks) and at 48 and 72 weeks. Error bars indicate interquartile range (IQR). Green arrows signify difference in vaccine dose at 24 weeks. **b.** Median ConM SOSIP.v7-specific IgG levels (ng/mL) per vaccine group over time. Error bars indicate IQR. Full dose group N = 13. Fractional (Fx) dose group N = 10. **C.** Median ConM SOSIP.v7-specific IgG levels (ng/mL) per sex at birth over time. Error bars indicate IQR. Female N = 13. Male N = 10. **d.** Median ConM SOSIP.v7-specific IgG levels (ng/mL) per sex at birth at 10, 26, 48 and 72 weeks. Error bars indicate IQR. Respective fold changes are indicated. Transgender individual indicated in grey. All figures represent the per-protocol cohort (N = 23). Differences between groups and sex were calculated by a two-tailed Mann-Whitney U-test. ** $p < 0.01$, ns = not significant. Source data are provided as a Source Data file.

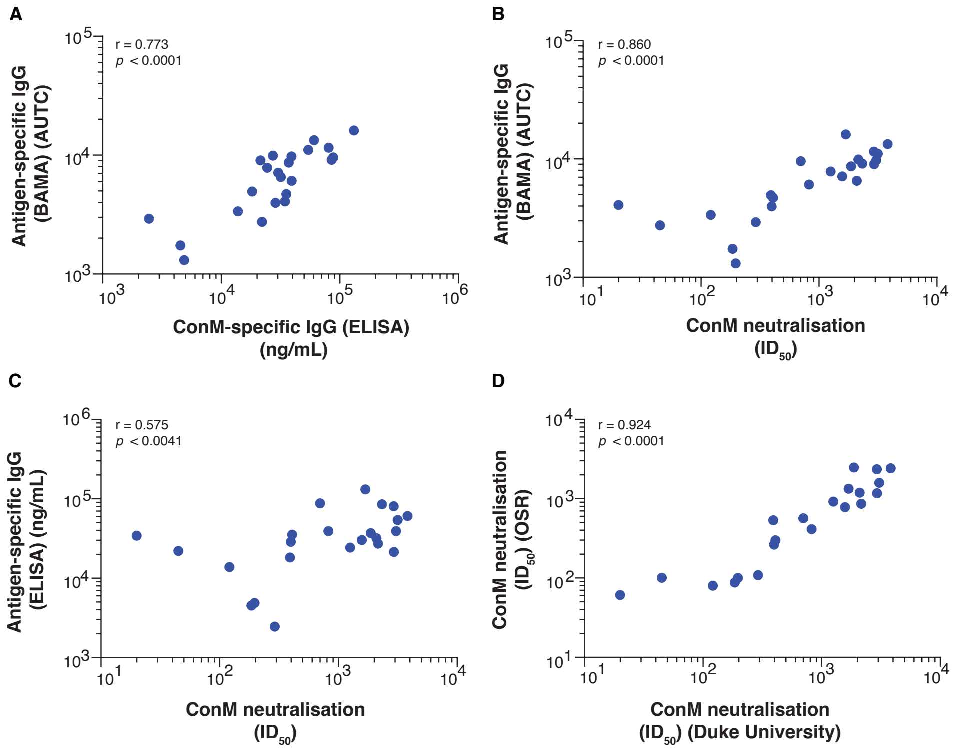

Supplementary Fig. 2. Correlation between serological assays. **a.** Correlation of median ConM SOSIP.v7-specific IgG measured by BAMA (AUTC) and ELISA (ng/mL) at 26 weeks. Spearman rho correlation coefficient (r) and *p*-value are shown. **b and c.** Correlation of median ConM SOSIP.v7-specific-IgG measured by BAMA (AUTC) and ELISA (ng/mL), respectively, and median ConM-pseudovirus serum neutralisation (ID_{50}) at 26 weeks. Spearman rho correlation coefficient (r) and *p*-value are shown. Data represents week 26 **d.** Correlation of median ConM-pseudovirus serum neutralisation (ID_{50}) measured by different laboratories at 26 weeks. OSR: Ospedale San Raffaele, Milan, Italy. Spearman rho correlation coefficient (r) and *p*-value are shown. All figures represent the per-protocol cohort (N = 23). Source data are provided as a Source Data file.

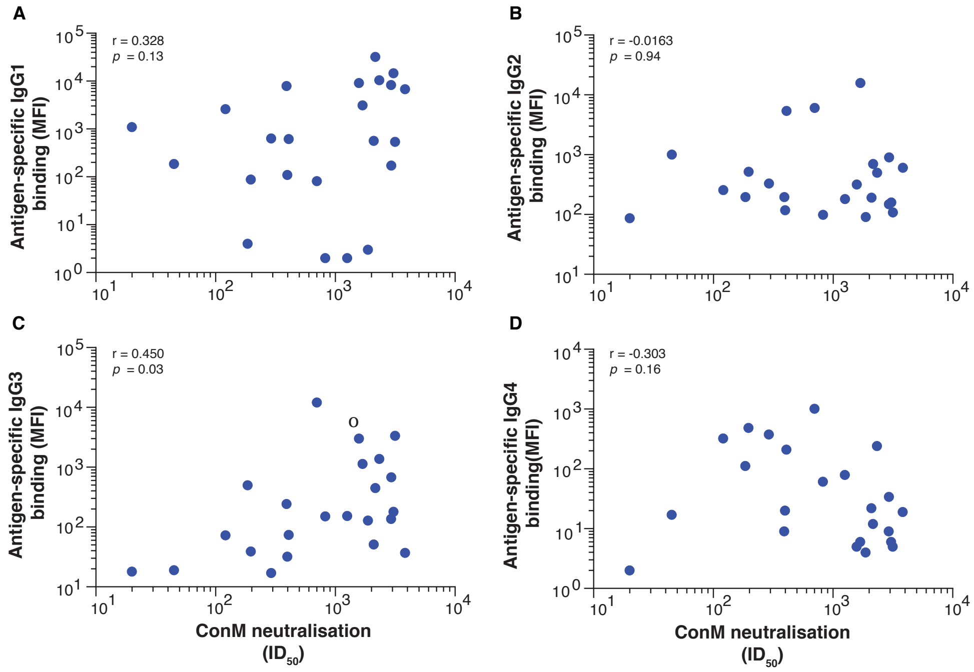

Supplementary Fig. 3. Correlation IgG subclasses and neutralisation. **a. – d.** Correlation of median ConM SOSIP.v7-specific IgG subclasses 1 – 4 (MFI) detected by Luminex and median ConM-pseudovirus serum neutralisation (ID_{50}) at 26 weeks. All figures represent the per-protocol cohort (N = 23). Spearman rho correlation coefficient (r) and *p*-value are shown. Source data are provided as a Source Data file.

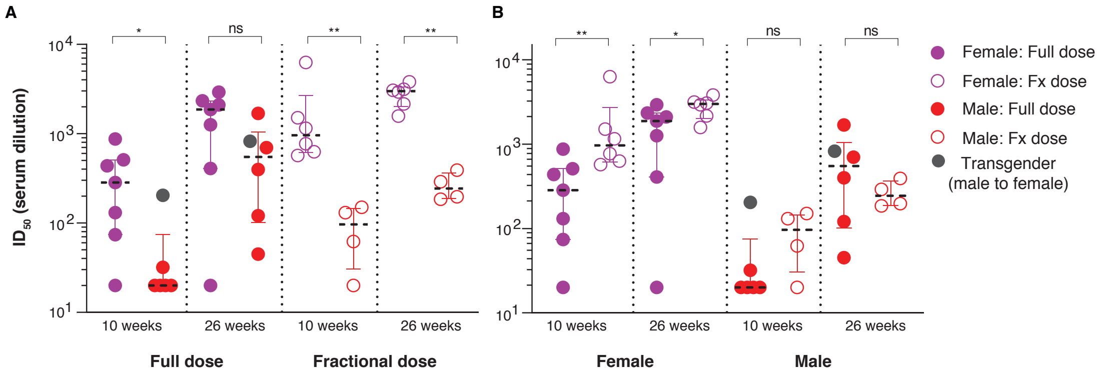

Supplementary Fig. 4. Serum neutralisation within group and sex. **a.** Median ConM-pseudovirus serum neutralisation titres (ID_{50}) per sex at birth, within vaccine groups at 10 and 26 weeks. Error bars indicate interquartile range (IQR). **b**. Median ConM-pseudovirus serum neutralisation titres (ID_{50}) per vaccine group, within females and male participants at 10 and 26 weeks. Error bars indicate IQR. All figures represent the per-protocol cohort (N = 23). Differences between groups were calculated by a two-tailed Mann-Whitney U-test. * $p < 0.05$, ** $p < 0.01$, ns = not significant. Source data are provided as a Source Data file.

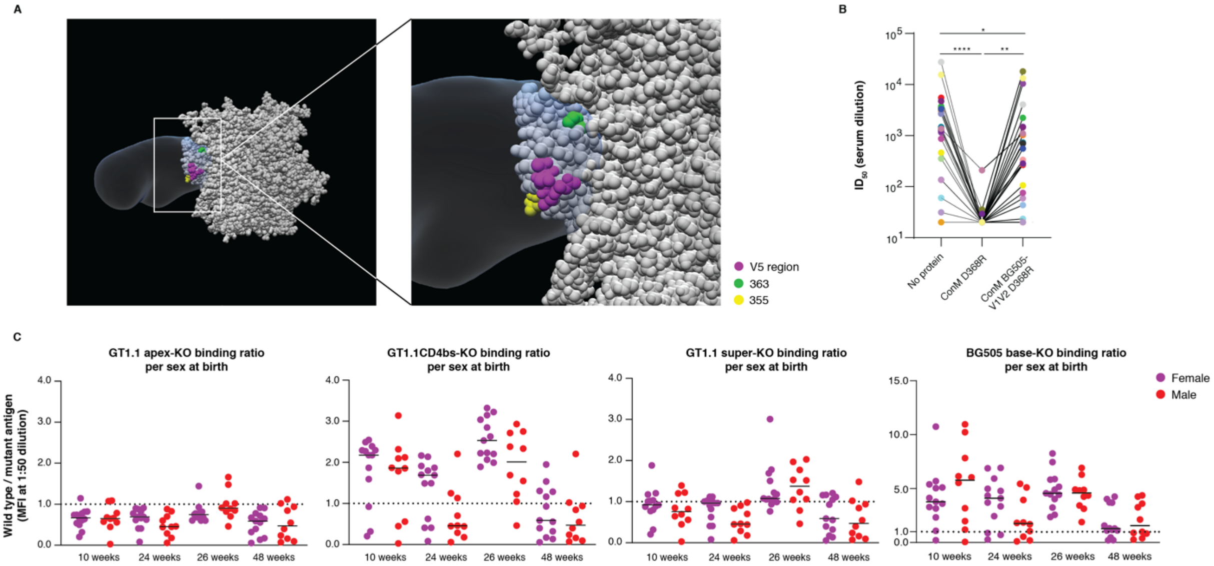

Supplementary Fig. 5. Epitope characterisation. **a.** C3V5-directed polyclonal EMPEM map from PubID 202 (see also **Fig. 5**) aligned to a crystal structure of ConM SOSIP.v7 with Env residues 355 (yellow), 363 (green) and V5 region (magenta). Position 355 is a PNGS in ConM. **b.** Serum neutralisation titres (ID_{50}) at 26 weeks against ConM in the presence or absence of ConM SOSIP.v7 D368R or ConM-BG505 V1V2 D368R, in which the V1V2 region was swapped for that of BG505. Statistical analysis was performed using a Friedman test followed by a Dunn's multiple comparison test. * $p < 0.05$, ** $p < 0.01$, **** $p < 0.0001$. **c**. Binding ratio of wild type antigen (BG505 SOSIP.GT1.1) to mutant (median fluorescence intensity (MFI) at 1:50 dilution). BG505 SOSIP.GT1.1 to BG505 SOSIPv8.1-GT1.1 apex-KO, BG505 SOSIPv4.1-GT1.1 CD4bs-KO and BG505 SOSIPv8-GT1.1 super-KO (apex and CD4bs epitope KO mutations combined); BG505 SOSIP.664 to ConM-BG505 V1V2 and BG505 SOSIP.v5 base-KO + 613T. Data is shown per sex at birth. Female N = 13. Male N = 10. Differences between sex were calculated by a two-tailed Mann-Whitney U-test. None of the comparisons were statistically significant ($p < 0.05$). Source data are provided as a Source Data file.

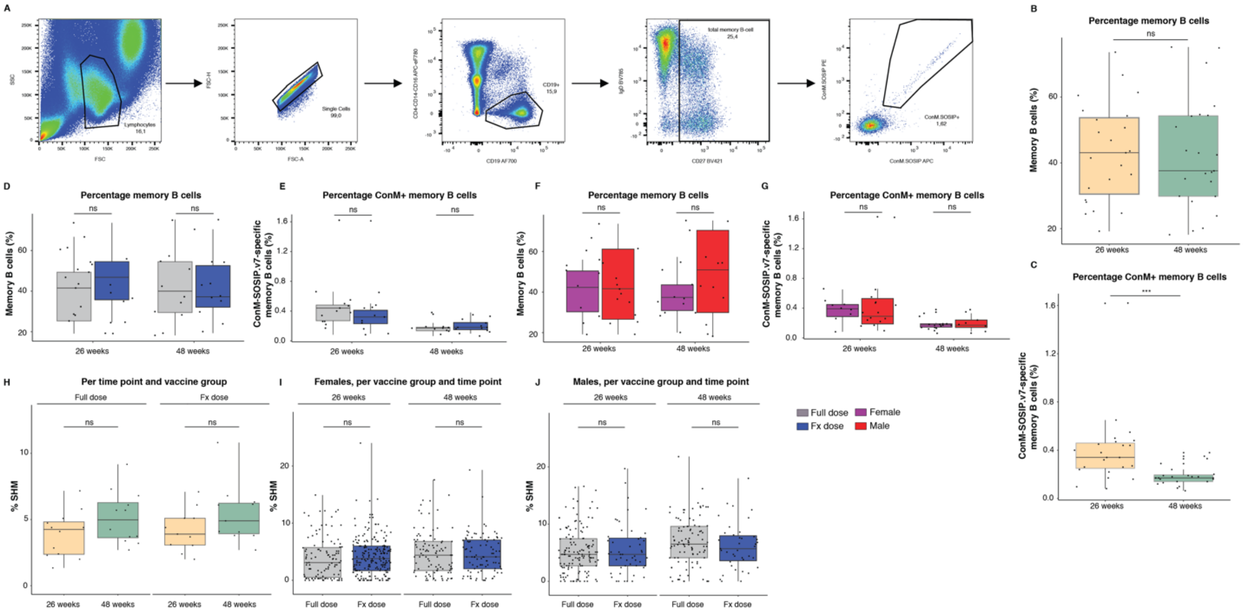

Supplementary Fig. 6. FACS strategy, ConM specific B cell frequencies and SHM. a. Example of FACS sorting strategy. Cells were gated on single cell lymphocytes from which viable CD19+ cells were gated. From the total B-cell compartment, total memory B cells were identified. Within this population, ConM SOSIP.v7+ cells were selected for sorting. **b.** Percentage of total memory B cells of the total B cell population, shown at 26 and 48 weeks. **c.** Percentage of ConM SOSIP.v7+ cells out of total memory B cells, shown at 26 and 48 weeks. Percentage of total memory B cells of the total B cell population, shown at 26 and 48 weeks, per **d.** vaccine group and **f.** sex at birth. Percentage of ConM SOSIP.v7+ cells out of total memory B cells, shown at 26 and 48 weeks, per **e.** vaccine group and **g.** sex at birth. V-gene somatic hypermutation (SHM) levels (%) within the ConM SOSIP.v7-specific memory B-cell population, measured at 26 weeks and 48 weeks per **h.** vaccine group (Full dose group N = 13, Fractional (Fx) group N = 10), **i.** female and **j.** male participants per vaccine group, per time point. Full dose female (N = 7), Fx dose female (N = 6), Full dose male (N = 6), Fx dose male (N = 4). Box plots indicate median SHM values, 25th and 75th percentiles; whiskers represent minima and maxima. Differences between groups and sex were calculated by Wilcoxon signed rank test, paired comparisons were calculated by donor median SHM two-sided paired Wilcoxon signed rank test. * $p < 0.05$, ** $p < 0.01$, *** $p < 0.001$, **** $p < 0.0001$, ns = not significant ($p > 0.05$). Source data are provided as a Source Data file.

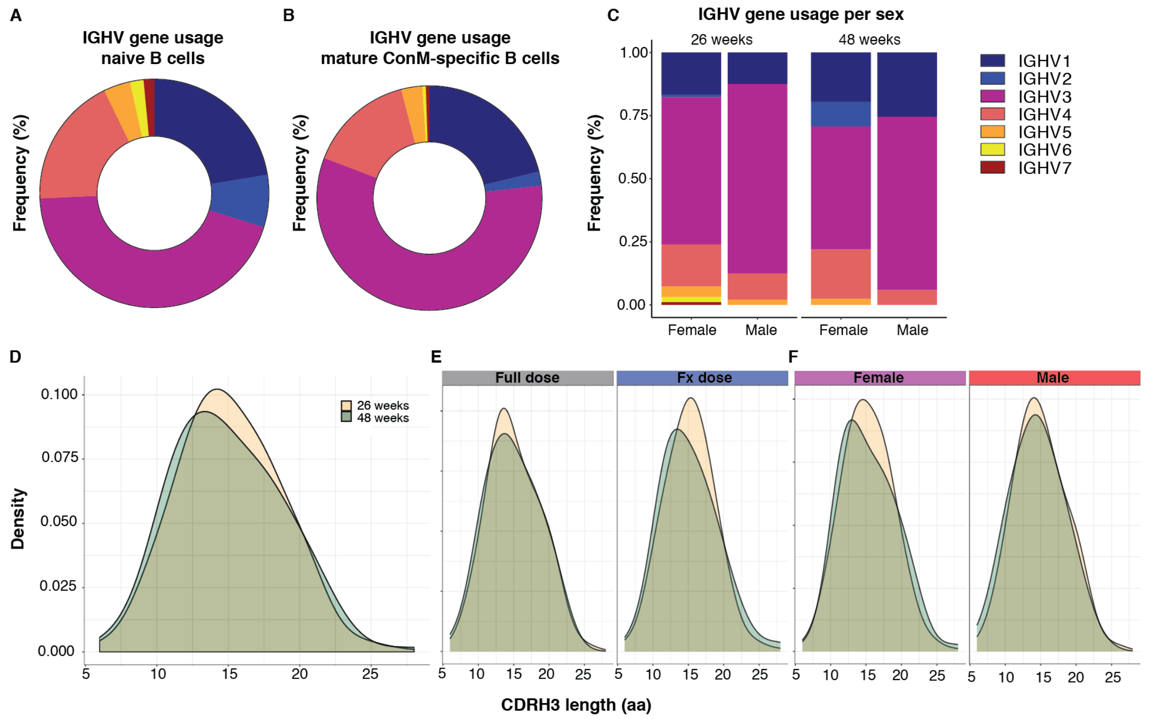

Supplementary Fig. 7. IGHV gene usage and mean CDRH3 length. **a.** IGHV gene usage in IgM repertoires of the total naïve B cell repertoire of each participant determined by IgDiscover, arranged by IGHV family (IGHV1 – IGHV7). **b.** HC V-gene usage for mature ConM SOSIP.v7-specific BCR sequences detected at weeks 26 and 48 (combined) was similarly mapped for the complete per protocol cohort (N = 23). **c.** HC V-gene usage for mature ConM SOSIP.v7-specific BCR sequences detected at weeks 26 and 48 (separated) per sex at birth. Female N = 13. Male N = 10. **d.** Mean CDRH3 length (in amino acids) for ConM SOSIP.v7-specific BCR sequences for all participants combined, at weeks 26 (beige) and 48 (green), **e.** per vaccine group and **f.** per sex at birth. Full dose group N = 13 (grey). Fractional (Fx) dose group N = 10 (blue). Female N = 13 (magenta). Male N = 10 (red). Source data are provided as a Source Data file.

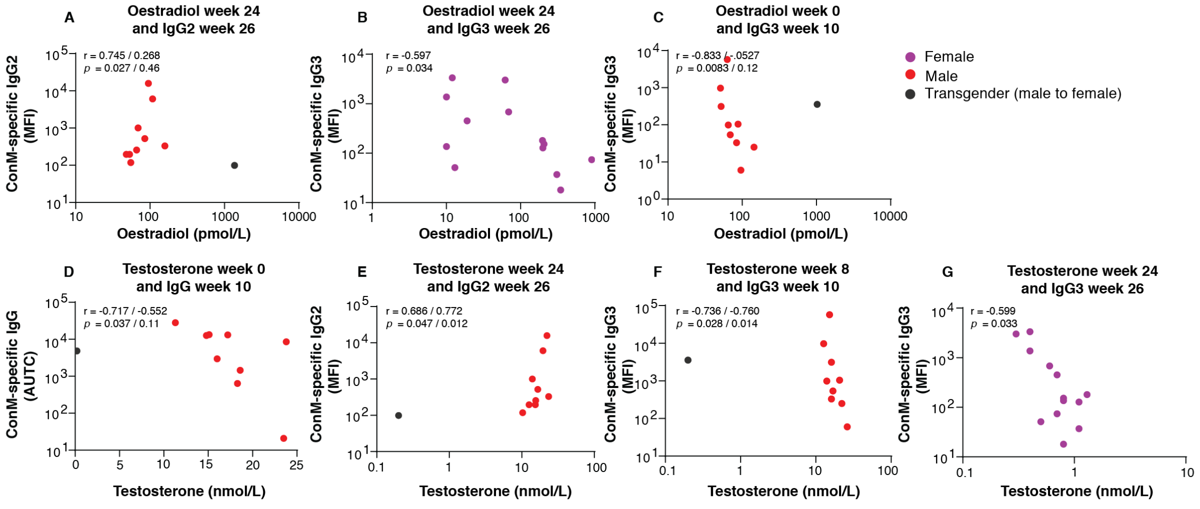

Supplementary Fig. 8. Correlations of sex steroid hormones and serological outcomes. a. – **g.** Plasma levels of oestradiol (pmol/L), progesterone (nmol/L) and testosterone (nmol/L) were measured at baseline, eight and 24 weeks. Graphs illustrate selected strongest Spearman correlations between hormone levels and ConM SOSIP.v7-specific IgG (AUTC) and IgG1-4 (MFI) at weeks 10 and 26, either for female (magenta, N = 13), male (red, N = 9) or transgender (MtF) (grey, N = 1) participants. Panels **a**, **c**, **d**, **e** and **f** show correlation coefficients and *p* values excluding and including transgender individual, respectively. *P*-values of < 0.004 were considered significant upon correcting for multiple testing (Bonferroni correction). Plasma oestradiol levels over time (weeks 0, 8 and 24) differed between female-born participants on oral contraceptives (median 54 pmol/L, range 10 – 460) and those not taking oral contraceptives (median 345 pmol/L, range 58 – 1707).

Plasma levels of oestradiol at 24 weeks correlated positively with IgG2 in male participants (Spearman r = 0.745, p = 0.027) and negatively with IgG3 in females at 26 weeks (Spearman r = -0.597, p = 0.034) (**a-b**). Oestradiol levels at day zero showed a strong negative correlation with antigen-specific IgG3 in male participants at 10 weeks (Spearman r = -0.833, p = 0.0083) (**c**). Testosterone at baseline showed a strong inverse correlation with total IgG in males at 10 weeks (Spearman r = -0.717, p = 0.037) (**d**). Testosterone levels at 24 weeks correlated positively with IgG2 at 26 weeks (Spearman r = 0.686, p = 0.047) (**e**), while increased testosterone at eight weeks correlated with lower levels of IgG3 at 10 weeks in male participants (Spearman r = -0.736, p = 0.028) (**f**). Testosterone levels at 24 weeks correlated inversely with IgG3 in female individuals at 26 weeks (Spearman r = -0.599, p = 0.033) (**g**). No correlations between progesterone and antigen-specific IgG and IgG subtypes were found. Source data are provided as a Source Data file.

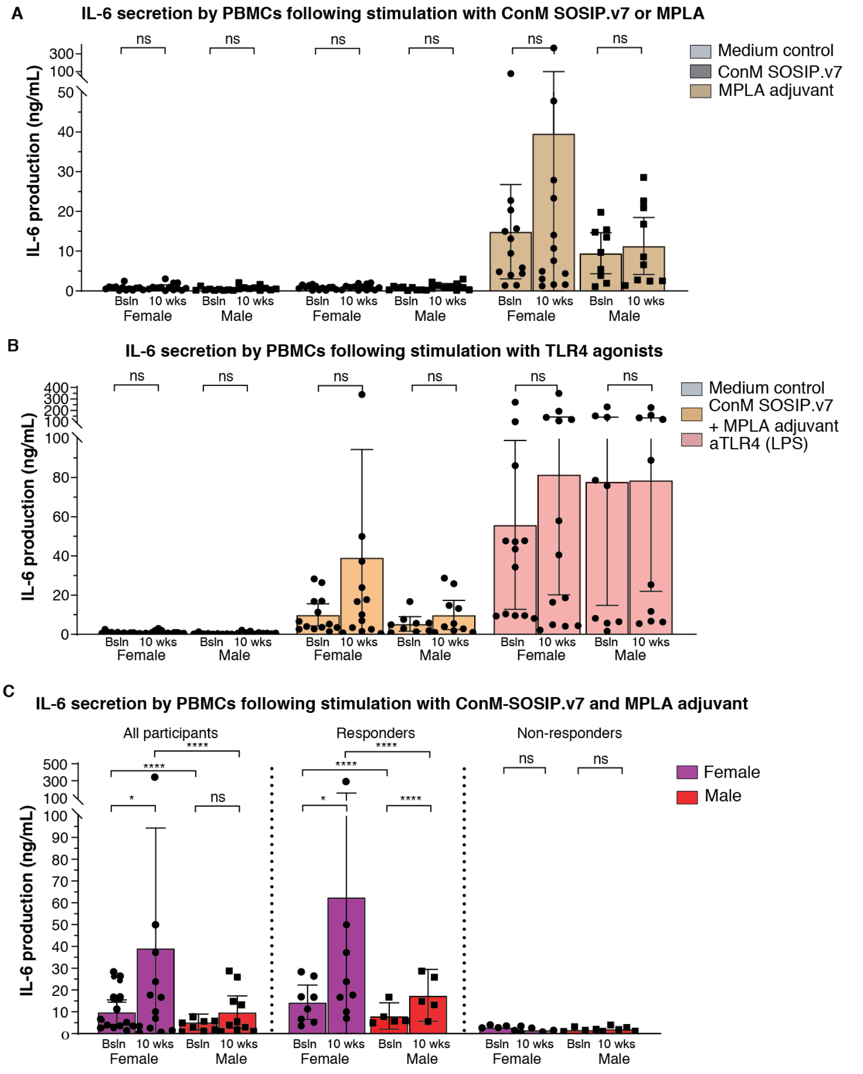

***Supplementary Fig. 9. IL-6 responses vary between females and males for MPLA*. a.** PBMCs were incubated for 24 hours with ConM SOSIP.v7 (5 µg/mL) or MPLA (30 µg/mL) for 24 hours after which SN was harvested. **b.** PBMCs were incubated for 24 hours with ConM SOSIP.v7 (5 µg/mL) and MPLA (30µg/mL) or bacterial Lipopolysaccharide (LPS, 10 ng/mL), a TLR4 ligand after which supernatant (SN) was harvested. IL-6 levels were subsequently determined in the SN using ELISA. No differences were detected when female and male PBMCs were stimulated with LPS, which also targets TLR4. However, this agonist demonstrated higher potency, which may have obscured subtle differences between female and male participants. **c.** Within both vaccine groups there were several non-responders, defined as having no IL-6 response (>5 ng/mL) following stimulation at both time points. IL-6 secretion for innate responders (middle) comprises N = 8 female participants and N = 5 male participants at both timepoints. Bsln = baseline. 10 wks = 10 weeks. Statistical testing was performed between both groups using two-way NOVA. All participants: * p = 0.030; Responders: * p = 0.024; **** p < 0.0001, ns = not significant. Source data are provided as a Source Data file.

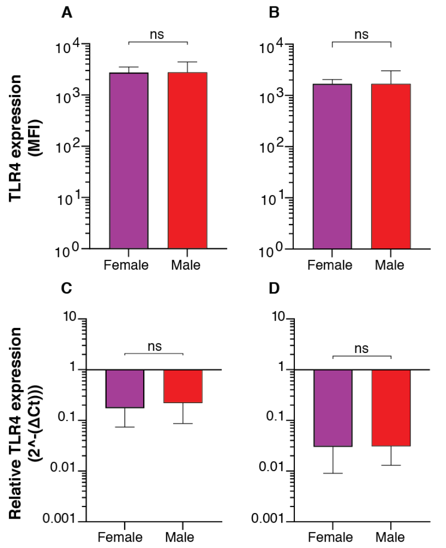

Supplementary Fig. 10. TLR4 expression levels did not differ between female and male participants. Absolute TLR4 expression levels on **a**. classical and **b**. CD16+ monocytes were measured by flowcytometry and expressed as mean fluorescence intensity (MFI). Median MFI and range are shown for female (N = 13) and male (N = 10) participants. **c**. TLR4 mRNA expression within total PBMC was quantified by qPCR. Expression relative to the GAPDH housekeeping gene is shown for female (N = 13) and male (N = 10) participants. **d**. Similar to panel **c**., corrected for the proportion (%) of monocytes within total PBMC. All analyses were performed on baseline (week -4) samples. Differences between groups were calculated by two-tailed Mann-Whitney U-test. ns = not significant. Source data are provided as a Source Data file.

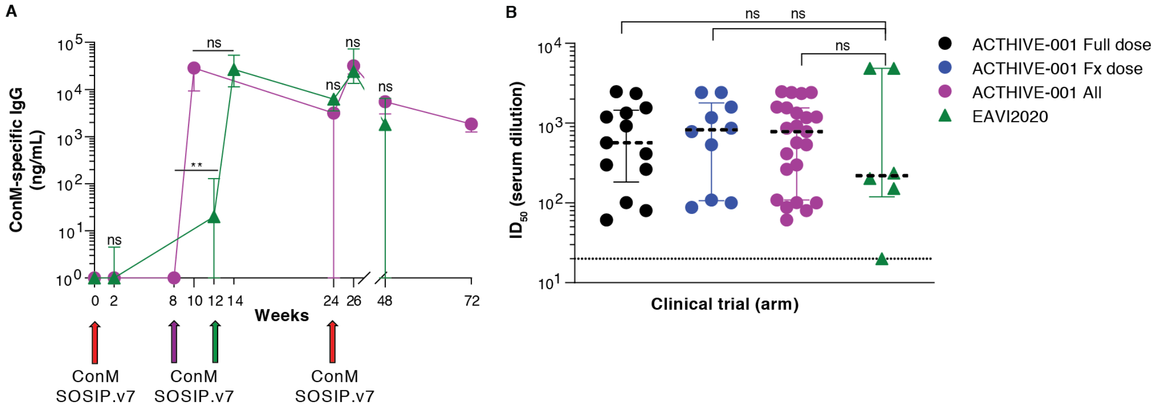

Supplementary Fig. 11. ConM SOSIP.v7-specific serological responses across different clinical studies. Participants in one arm of the EAVI2020 trial received an identical dosage (100 µg) of ConM SOSIP.v7, adjuvanted with (500 µg of) MPLA liposomes at baseline, 12 and 24 weeks. **a.** ConM SOSIP.v7-specific IgG levels measured by enzyme-linked immuno sorbent assay (ELISA) for the ACTHIVE-001 (N = 23) and EAVI2020 clinical trials (N = 10). Assays performed at consortium laboratory at Imperial College London. Values indicate median IgG levels (ng/mL) and are depicted at each vaccination baseline (zero, 8 and 24 weeks for ACTHIVE-001; zero, 12 and 24 weeks for EAVI2020), two weeks post-vaccination and at 48 and 72 weeks (ACTHIVE-001 only). **b.** ConM-pseudovirus serum neutralisation. 50% inhibitor dilution (ID_{50}) values are depicted two weeks post third vaccination (26 weeks) for the ACTHIVE-001 Full dose group (N = 13) and Fractional (Fx) dose group (N = 10), total ACTHIVE-001 cohort (N = 23) and EAVI2020 (N = 6). Values are the serum dilution at which relative luminescence units (RLUs) were reduced 50% compared to virus control wells. Assays performed at consortium laboratory at Ospedale San Raffaele. Differences between groups were calculated by two-tailed Mann-Whitney U-test. ** $p < 0.01$, ns = not significant. EAVI2020 vaccine recipients (N = 10) showed a more rapid antibody binding response following the first vaccination, with two out of 10 participants having low yet detectable levels of antigen-specific IgG (**a**), while no response could be detected in ELISA for ACTHIVE-001 participants at this time. Some level of antigen-specific IgG could be picked up in ACTHIVE-001 participants using BAMA, but this assay was not performed on EAVI2020 samples. No significant differences in response magnitude were detected following the second and third vaccination (**a**). Similar outcomes were seen for autologous serum neutralisation following the full vaccination series at 26 weeks (**b**). Source data are provided as a Source Data file.

Supplementary Tables

	Vaccine group	
Post any vaccine dose<br>*Participants who reported:*	Full dose<br>(N = 13)	Fx dose<br>(N = 11)
at least one AR, n (%);	13 (100)	11 (100)
at least one local AR, n (%);	12 (92.3)	10 (90.9)
at least one local AR with severity Grade 3 as worst grade, n (%);	0 (0.0)	1 (9.1)
at least one systemic AR, n (%);	11 (84.6)	11 (100)
at least one systemic AR with severity Grade 3 as worst grade, n (%);	3 (23.1)	0 (0)
at least one AR leading to permanent stop of vaccine, n (%).	0 (0.0)	1 (9.1)

Supplementary Table 1. Solicited adverse reactions after any vaccination. N = Total number of participants in the safety analysis group. n = Cells represent the number of participants with an event of a specified category. % = Percentage of participants in each category, i.e., 100 x n/N. Fx = Fractional. Solicited adverse reactions (AR) are reported for the full duration of the study. Adverse events (AE) considered possibly, probably or definitely related to the vaccine were determined ARs. Source data are provided as a Source Data file.

	Vaccine group	
Post third vaccine dose<br>*Participants who reported:*	Full dose<br>(N = 13)	Fx dose<br>(N = 10)
at least one AR, n (%);	12 (92.3)	10 (100)
at least one local AR, n (%);	10 (76.9)	10 (100)
at least one systemic AR, n (%);	8 (61.5)	6 (60.0)
at least one AR with maximum severity Grade 3, n (%).	2 (15.4)	1 (10.0)

Supplementary Table 2. Solicited adverse reactions after third vaccination. N = Total number of participants in the safety analysis group who received the third vaccination. n = Cells represent the number of participants with an event of a specified category. % = Percentage of participants in each category, i.e., 100 x n/N. Fx = Fractional. Solicited adverse reactions (AR) are reported from immediately after the third vaccination until the final study visit. Adverse events (AE) considered possibly, probably or definitely related to the vaccine were determined ARs. Source data are provided as a Source Data file.

	Sex at birth	
Post any vaccine dose<br>*Participants who reported:*	Female<br>(N = 14)	Male*<br>(N = 10)
at least one AR, n (%);	14 (100)	10 (100)
at least one local AR, n (%);	12 (85.7)	9 (90)
at least one systemic AR, n (%);	13 (92.9)	9 (90)
at least one AR with maximum severity Grade 3, n (%).	1 (7.1)	3 (30.0)

Supplementary Table 3. Solicited adverse reaction distribution according to sex at birth. N = Total number of participants in the safety analysis group. n = Cells represent the number of participants with an event of a specified category. % = Percentage of participants in each category, i.e., 100 x n/N. Solicited adverse reactions (AR) are reported for the full duration of the study. Adverse events (AE) considered possibly, probably or definitely related to the vaccine were determined ARs. * One participant was male at birth, has undergone gender-affirming surgery and currently uses feminising hormone therapy. Source data are provided as a Source Data file.

Vaccine group	Sex	Preferred term	Post dose	Post-dose period	Relation to vaccine (AR)	Outcome	Duration (days)
Fx dose	F	Fever	2	28 days	Unrelated	Resolved	2
Full dose	M	Malaise	3	7 days	Related	Resolved	1
Full dose	M	Headache	3	7 days	Related	Resolved	1
Full dose	M	Fever	1	7 days	Related	Resolved	1
Full dose	M	Panic attack	3	28 days	Unrelated	Resolved	2
Fx dose	F	Injection site erythema	2	7 days	Related	Resolved	2
Full dose	M	Fever	3	7 days	Related	Resolved	1
Fx dose	F	Injection site erythema	3	7 days	Related	Resolved	4

Supplementary Table 4. Solicited adverse events with maximum severity Grade 3. Solicited adverse events (AE) with maximum severity Grade 3 are reported for the full duration of the study. All AEs with maximum severity Grade 3 were reported within the seven- or 28-day post-vaccination period. AEs considered possibly, probably or definitely related to the vaccine were determined adverse reactions (AR). Fx = Fractional. Sex: sex at birth. F = female. M = male. Duration: number of days with maximum severity Grade 3. Duration includes end-date. Source data are provided as a Source Data file.

	Any n (%)	Grade 1 n (%)	Grade 2 n (%)	Grade 3 n (%)
Local adverse events				
Injection site tenderness	20 (83.3)	20 (83.3)	0 (0.0)	0 (0.0)
Injection site erythema	11 (45.8)	8 (33.3)	5 (20.8)	1 (4.2)
Injection site pain	7 (29.2)	7 (29.2)	0 (0.0)	0 (0.0)
Axillary pain (vaccinated arm)	3 (12.5)	3 (12.5)	0 (0.0)	0 (0.0)
Injection site swelling	3 (12.5)	2 (8.3)	1 (4.2)	0 (0.0)
Injection site pruritus	2 (8.3)	2 (8.3)	1 (42)	0 (0.0)
Injection site bruising	2 (8.3)	2 (8.3)	0 (0.0)	0 (0.0)
Stiffness and/or pain vaccinated arm	2 (8.3)	1 (4.2)	1 (4.2)	0 (0.0)
Confluent erythematous skin lesions (not injection site)	1 (4.2)	0 (4.2)	1 (4.2)	0 (0.0)
Systemic adverse events				
Headache	14 (58.3)	14 (58.3)	0 (0.0)	1 (4.2)
Chills	11 (45.8)	8 (33.3)	2 (8.3)	1 (4.2)
Malaise	11 (45.8)	9 (37.5)	3 (12.5)	0 (0.0)
Fatigue	8 (33.3)	8 (33.3)	2 (8.3)	0 (0.0)
Myalgia	7 (29.2)	7 (29.2)	1 (4.2)	0 (0.0)
Nausea	7 (29.2)	6 (25.0)	1 (4.2)	0 (0.0)
Fever	7 (29.2)	4 (16.7)	0 (0.0)	3 (12.5)
Pruritus (not injection site)	4 (16.7)	2 (8.3)	2 (8.3)	0 (0.0)
Abdominal pain	3 (12.5)	3 (12.5)	0 (0.0)	0 (0.0)
Sweating	3 (12.5)	3 (12.5)	0 (0.0)	0 (0.0)
Rash	2 (8.3)	2 (8.3)	0 (0.0)	0 (0.0)
Polydipsia	1 (4.2)	2 (8.3)	0 (0.0)	0 (0.0)
Suspected angioedema	1 (4.2)	1 (4.2)	0 (0.0)	0 (0.0)
Other adverse events				
Respiratory: coughing, sore throat, Influenza like symptoms	5 (20)	0 (0.0)	0 (0.0)	0 (0.0)

Supplementary Table 5. Solicited adverse reactions per symptom class by maximum reported severity. n = Cells represent the number of participants with an event of a specified category. % = Percentage of participants in each category, based on the total of number of participants in the safety analysis group (N = 24). Adverse events (AE) considered possibly, probably or definitely related to the vaccine were determined adverse reactions (AR). Solicited adverse reactions are reported in the following symptom classes: local, systemic, and other. Within the '*Other adverse reactions*' category, only respiratory ARs were reported. Source data are provided as a Source Data file.

Supplementary Methods

In- and exclusion criteria

In order to be eligible to participate in this study, a subject had to meet all of the (applicable) inclusion criteria and could not meet any of the (applicable) exclusion criteria.

Inclusion criteria

1. Men and women, aged between 18 and 50 years on the day of screening.

2. Willing to comply with the requirements of the protocol and available for follow-up for the planned duration of the study.

3. Willing and able to give written informed consent.

4. Willing to undergo HIV testing, risk reduction counselling and receive HIV test results, including the possibility of vaccine-induced seropositivity (VISP).

5. All female individuals engaging in sexual activity that could lead to pregnancy must commit to use an effective method of contraception for four months following Investigational Medicinal Product administration, including:
    - Condoms (male or female) with or without spermicide
    - Diaphragm or cervical cap with spermicide
    - Intrauterine device, or contraceptive implant
    - (Oral) hormonal contraception
    - Successful vasectomy in the male partner (considered successful if a woman reports that a male partner has [1] documentation of azoospermia by microscopy (< 1 year ago), or [2] a vasectomy more than two years ago with no resultant pregnancy despite sexual activity post-vasectomy)
    - Not be of reproductive potential, such as having undergone hysterectomy, bilateral oophorectomy, or tubal ligation, postmenopausal (> 45 years of age with amenorrhea for at least two years, or any age with amenorrhea for at least 6 months and a serum follicle stimulating hormone [FSH] level > 40 IU/L); surgically sterile: no additional contraception required

6. All female volunteers who are not heterosexually active at screening, must agree to utilise an effective method of contraception if they become heterosexually active, as outlined above.

7. All female volunteers must be willing to undergo urine pregnancy tests at time points indicated in the Schedule of Visits and Procedures (Study Protocol Appendix 1).

8. All sexually active male volunteers, regardless of reproductive potential, must be willing to use an effective method of contraception (such as consistent condom use) from the day of first vaccination until

at least four months after the last vaccination to avoid exposure of partners to Investigational Medicinal Product in ejaculate and to prevent conception with female partners.

9. Willing to abstain from donating blood, eggs or sperm from the day of first vaccination until at least 3 months after the end of their participation in the trial and, for those who test HIV-positive due to vaccine-induced antibodies, until the anti-HIV antibody titres become undetectable

10. All volunteers must be registered with a general practitioner.

Exclusion criteria

1. Confirmed HIV-1 or HIV-2 infection (HIV Ag/Ab plus HIV RNA testing)

2. Self-reported risk for HIV exposure or STIs prior to screening, defined as:

    - Unprotected sexual intercourse with a known HIV infected person, a partner known to be at high risk for HIV infection or a casual partner (i.e., no continuing established relationship) in the last six months. *Note:* Individuals engaging in unprotected sexual intercourse that use pre-exposure prophylaxis (PrEP) in an adequate manner (as judged by the principal investigator, following the Dutch PrEP guideline), either on demand or daily, can be enrolled in the study.
    - Engaged in sex work in the last twelve months
    - Frequent excessive daily alcohol use or frequent binge drinking, or any use of illicit drugs in the last twelve months
    - Self-reported history of newly-acquired syphilis, gonorrhoea, non-gonococcal urethritis, HSV-2, chlamydia, pelvic inflammatory disease (PID), trichomonas, mucopurulent cervicitis, epididymitis, proctitis, lymphogranuloma venereum, chancroid, or hepatitis B in the last twelve months
    - MSM or transgender persons having had unprotected anal intercourse in the last six months with either a male partner with an unknown HIV status OR a HIV positive partner with a (presumed) detectable viral load
    - MSM or transgender persons diagnosed with a rectal STI in the last six months
    - MSM or transgender persons who have been prescribed post-exposure prohylaxis (PEP) in the last six months

3. If female, pregnant or planning a pregnancy during the period of enrolment until four months after the last study vaccination; or lactating.

4. Any clinically relevant medical condition that is considered in the opinion of the investigator to make the volunteer unsuitable for participation in the study (under which underlying haematological disorders, auto-immune disease, immunodeficiency, gastrointestinal, hepatic and cardiopulmonary disorders). This also includes a history of malignancy in the past five years (prior to screening) or ongoing malignancy. (Note: A history of a completely excised malignancy that is considered cured is not an exclusion).

5. Infectious disease in the six months before screening: acute and chronic hepatitis B infection (HbsAg-positive), hepatitis C infection (anti-HCV and HCV RNA positive), treatment for chronic hepatitis C infection in the past year, or active syphilis (positive chemiluminescence immunoassay (LIAISON XL), confirmed by positive RPR).

6. History of hyposplenia (anatomical or functional).

7. Bleeding disorder that was diagnosed by a physician (e.g., factor deficiency, coagulopathy or platelet disorder that requires special precautions.) (Note: A volunteer who states that he or she has easy bruising

or bleeding, but does not have a formal diagnosis and has intramuscular injections and blood draws without any adverse experience, is eligible).

8. Receipt of live attenuated vaccine within the previous 30 days or planned receipt within 30 days after IP administration; or receipt of other vaccine within the previous 14 days or planned receipt within 14 days after IP administration. (Exception is live attenuated influenza vaccine within 14 days.)

9. Receipt of blood products or blood-derived products within four months of screening.

10. Participation in another clinical trial of an Investigational Medicinal Product currently, within the previous three months or expected participation during this study. Concurrent participation in an observational study, not involving medicinal products and not requiring any blood or tissue sample collection is not an exclusion criterion.

11. Prior receipt of another investigational HIV vaccine or HIV monoclonal antibody (product). (Note: receipt of placebo in a previous HIV vaccine trial will not exclude a volunteer from participation if documentation is available.)

12. Known hypersensitivity to any component of the vaccine formulation used in this trial, or severe or multiple allergies to drugs or pharmaceutical agents.

13. Positive reaction in antinuclear antibody (ANA) screen and positive reaction in subsequent anti-dsDNA or anti-ENA assessment; or clinically significant immunoglobulin (IgA, IgG or IgM) values.

14. Use of any medications, including over-the-counter products, which, in the opinion of the investigators, would either interfere with the study or potentially cause harm to the volunteer. Use of corticosteroids, immunosuppressants, chemotherapeutics, anti-tuberculosis or other medications considered significant by the investigator within the previous six months. The following exceptions are permitted and will not exclude study participation: use of corticosteroid nasal spray for rhinitis, topical corticosteroids for an acute uncomplicated dermatitis (except for steroids applied to the non-dominant upper arm); or a short course (duration of ten days or less, or a single injection) of corticosteroid for a non-chronic condition (based on investigator clinical judgment) at least two weeks prior to enrolment in this study.

15. Unable to read and speak Dutch or English to a fluency level adequate for the full comprehension of procedures required in participation and consent.

16. Active, serious infections requiring (par)enteral antibiotic, antiviral or antifungal therapy within 30 days prior to enrolment.

17. Seizure disorder: A participant who has had a seizure in the last three years prior to screening is excluded. (Not excluded: a participant with a history of seizures who has neither required medications nor had a seizure for three years).

18. Grade ≥ 1 clinically significant routine safety laboratory parameters.

19. If, in the opinion of the Principal Investigator, it is not in the best interest of the volunteer to participate in the trial.

20. Body mass index (BMI) ≥ 35 kg/m^2 or body weight < 50 kg.

Primary and secondary study outcomes

The primary and secondary study outcomes as per the study protocol are listed below. Not all outcomes have been included in this work. Outcomes included in this work are indicated with an asterisk (*).

Primary study endpoints

Safety and tolerability:

1. Proportion of volunteers with a ≥ grade 3 adverse event, from the day of each vaccination up to 7 days post each vaccination (*).

2. Proportion of volunteers with ≥ grade 3 and/or vaccine related adverse events, including safety laboratory (biochemical, haematological) parameters, from the day of each vaccination up to 28 days post each vaccination (*).

3. Proportion of volunteers with vaccine-related serious adverse events throughout the study period (*).

Secondary study endpoints

Immunogenicity:

1. Autologous neutralising antibodies induced by ConM SOSIP.v7 gp140 vaccine, adjuvanted in MPLA liposomes:
    a. Serum titres of autologous neutralising antibodies (*);
    b. Proportion of volunteers with autologous neutralising antibodies (*).

2. Trimer binding antibody responses induced by ConM SOSIP.v7 gp140 vaccine, adjuvanted in MPLA liposomes:
    a. Magnitude of the trimer binding antibody response (*);
    b. Proportion of volunteers with a trimer binding antibody response (*).

3. Heterologous neutralising antibodies induced by ConM SOSIP.v7 gp140 vaccine, adjuvanted in MPLA liposomes:
    a. Serum titres of heterologous neutralising antibodies (i.e., against additional (Tier 1a/b, Tier 2) virus strains (*));
    b. Proportion of volunteers with heterologous neutralising antibodies (*).

4. Induction of Env-specific B cell responses will be analysed and comparisons made between individuals with low, high and durable NAb titres (i.e., persistent at 12- and 18-months follow-up):

    - Env-specific plasmablast repertoire sequence analysis in peripheral blood;

    - Env-specific germline and memory B cell repertoire sequence analysis in peripheral blood (*);

    - Env-specific germinal centre B cell repertoire sequence analysis in the draining lymph node(s).

Exploratory study parameters/endpoints

Immunogenicity:

1. Proportion of volunteers with and magnitude of binding antibodies to other HIV-1 Env proteins (e.g., subtypes A, B, C) (*).

2. Frequency of HIV-1 Env specific B and T(fh) cells

3. Characteristics of Env-specific monoclonal antibodies (IgG) from isolated memory B cells and plasmablasts (the latter sorted according to homing receptors)

4. Breadth and epitope specificity of HIV-1 Env-specific polyclonal antibodies (pAb) (*)

5. Affinity of polyclonal antibodies in serum and ConM SOSIP.v7 gp140, quantified by biolayer interferometry (Octet)

6. Transcriptional profile of innate and adaptive immune responses

7. Frequency and characteristics of antibody effector functions (e.g., ADCC/ADCP-mediating antibodies)

8. Frequency of HIV-1 Env-specific T(fh) cells after vaccination in the lymphoid compartment (lymph node FNA)
