# Supplementary material for: HIV-1 envelope trimer vaccine induces sex-associated differences in antibody responses: a phase 1 clinical trial
Source: Nat Commun. 2025 Nov 21;16:10250. doi: 10.1038/s41467-025-65101-7 (PMC12639139; doi:10.1038/s41467-025-65101-7)
Supplement: Supplementary file 2 — Reporting Summary [file 41467_2025_65101_MOESM2_ESM.pdf]

nature portfolio

Corresponding author(s): Emma I.M.M. Reiss

Last updated by author(s): Aug 22, 2025

Reporting Summary

Nature Portfolio wishes to improve the reproducibility of the work that we publish. This form provides structure for consistency and transparency in reporting. For further information on Nature Portfolio policies, see our Editorial Policies and the Editorial Policy Checklist.

Statistics

For all statistical analyses, confirm that the following items are present in the figure legend, table legend, main text, or Methods section.

n/a	Confirmed	
☐	☒	The exact sample size (*n*) for each experimental group/condition, given as a discrete number and unit of measurement
☐	☒	A statement on whether measurements were taken from distinct samples or whether the same sample was measured repeatedly
☐	☒	The statistical test(s) used AND whether they are one- or two-sided<br>*Only common tests should be described solely by name; describe more complex techniques in the Methods section.*
☐	☒	A description of all covariates tested
☐	☒	A description of any assumptions or corrections, such as tests of normality and adjustment for multiple comparisons
☐	☒	A full description of the statistical parameters including central tendency (e.g. means) or other basic estimates (e.g. regression coefficient) AND variation (e.g. standard deviation) or associated estimates of uncertainty (e.g. confidence intervals)
☒	☐	For null hypothesis testing, the test statistic (e.g. *F*, *t*, *r*) with confidence intervals, effect sizes, degrees of freedom and *P* value noted<br>*Give P values as exact values whenever suitable.*
☒	☐	For Bayesian analysis, information on the choice of priors and Markov chain Monte Carlo settings
☒	☐	For hierarchical and complex designs, identification of the appropriate level for tests and full reporting of outcomes
☐	☒	Estimates of effect sizes (e.g. Cohen's *d*, Pearson's *r*), indicating how they were calculated

Our web collection on statistics for biologists contains articles on many of the points above.

Software and code

Policy information about availability of computer code

Data collection	Clinical data was collected at study visits and entered into the electronic participant file. Data was transferred manually to the Castor EDC database and checked by multiple study staff members. After study close-out, safety data was exported and processed in Microsoft Excel version 16.54. The collection of samples and initial processing is described in the Methods section. No computer code was used for data collection.
Data analysis	The majority of analyses were performed in Graphpad version Prism 9.3.1, in addition to software used as stated in the Methods section. No computer code was used for data analysis.

For manuscripts utilizing custom algorithms or software that are central to the research but not yet described in published literature, software must be made available to editors and reviewers. We strongly encourage code deposition in a community repository (e.g. GitHub). See the Nature Portfolio guidelines for submitting code & software for further information.

Data

Policy information about availability of data

All manuscripts must include a data availability statement. This statement should provide the following information, where applicable:

- Accession codes, unique identifiers, or web links for publicly available datasets
- A description of any restrictions on data availability
- For clinical datasets or third party data, please ensure that the statement adheres to our policy

The data availability statement includes all necessary accession codes and web links.

Human research participants

Policy information about studies involving human research participants and Sex and Gender in Research.

Reporting on sex and gender	Information on gender and sex was collected during the screening visit upon acquiring Informed Consent. Findings were reported according to sex at birth, as is indicated throughout the manuscript.
Population characteristics	The target population consisted of HIV-uninfected adult women and men in good general health, between the ages of 18 and 50 years. Considerable risk of exposition to HIV was avoided in order to minimise interference of exposition on induction of immune responses by the Investigational Medicinal Product. In order to be eligible to participate in this study, a participant had to meet all of the (applicable) inclusion criteria and could not meet any of the (applicable) exclusion criteria (listed in the Study Protocol).
Recruitment	Participants were recruited through information presented on the (online) recruitment platform 'www.proefpersonen.nl'. If interested, participants were sent a digital copy of the participant information sheet via e-mail. After reading they were asked to contact the study physician to complete a short interview by telephone to assess their eligibility for enrolment. During this preliminary assessment participants were able to ask initial questions. If eligible, participants were subsequently invited for an interview in person. This interview was conducted by the study physician and included going over the participant information sheet and Informed Consent Form. The trial was be discussed in detail (i.e. explaining of the aims, methods, benefits and potential hazards) and any questions about the study were answered. If participants were willing and interested they were asked to sign the Informed Consent Form.
Ethics oversight	This study received approval from the Central Committee on Research Involving Human Subjects, the Ministry of Health, Welfare and Sports of the Netherlands and the Medical Research Ethics Committee of the Amsterdam University Medical Centers (previously 'Academic Medical Center'). The clinical trial is registered at ClinicalTrials.gov under identification number NCT03961438.

Note that full information on the approval of the study protocol must also be provided in the manuscript.

Field-specific reporting

Please select the one below that is the best fit for your research. If you are not sure, read the appropriate sections before making your selection.

[x] Life sciences [ ] Behavioural & social sciences [ ] Ecological, evolutionary & environmental sciences

For a reference copy of the document with all sections, see nature.com/documents/nr-reporting-summary-flat.pdf

Life sciences study design

All studies must disclose on these points even when the disclosure is negative.

Sample size	A total of 20 participants needed to be enrolled. This number was selected based on comparable phase 1 HIV vaccine trials and the notion that this size is generally considered appropriate for a phase 1 clinical trial with a novel Investigational Medicinal Product. To account for up to 20% dropout after enrolment, an over-enrolment of two participants per vaccine group were permitted. The (statistical) implications of this sample size for the main safety endpoint (i.e., proportion of volunteers with a solicited grade ≥ 3 adverse event) and main immunogenicity endpoint (i.e., titre of serum neutralising antibodies) are described in Section 4.4 of the Study Protocol.
Data exclusions	Safety data were calculated on a modified inention-to-treat basis: participants who received at least one vaccination) (N = 24). Immunogenicity data were calculated for the per-protocol cohort: participants who received all three vaccinations and completed at least the 48 weeks follow-up visit (N = 23).
Replication	All clinical and experimental procedures and assays were documented in the Study Protocol, Analytical Plan and (local) Standard Operating Procedures. Clinical activities were carefully monitored by the independent study monitor. Safety data was collected in the electronic participant dossier and exported to an electronic database (Castor EDC) and a separate safety line listing file, which were cross-checked by multiple study staff members after study close-out. For the main immunological endpoint (serum binding and neutralising antibodies), findings were confirmed by two separate expert laboratories.

Randomization	Following enrolment, eligible participants were be block-randomised to one of the two vaccine groups, using a computer-generated algorithm (ALEA) with a set block size of two.
Blinding	Given the experimental nature of this small scale phase 1 study, only the laboratory teams undertaking immunological analysis were blinded and not the clinical staff or the study participants. Investigators performing subsequent analyses were likewise unblinded.

Reporting for specific materials, systems and methods

We require information from authors about some types of materials, experimental systems and methods used in many studies. Here, indicate whether each material, system or method listed is relevant to your study. If you are not sure if a list item applies to your research, read the appropriate section before selecting a response.

Materials & experimental systems

n/a	Involved in the study	
☐	☒	Antibodies
☐	☒	Eukaryotic cell lines
☒	☐	Palaeontology and archaeology
☒	☐	Animals and other organisms
☐	☒	Clinical data
☒	☐	Dual use research of concern

Methods

n/a	Involved in the study	
☒	☐	ChIP-seq
☐	☒	Flow cytometry
☒	☐	MRI-based neuroimaging

Antibodies

Antibodies used	Monoclonal antibodies (mAbs) used in BAMA experiments are listed in the Methods section. All mAbs except for RM19R and RM20A2 were provided (non-commercially) by the Protein Production Facility at the Duke University Human Vaccine Institute, Durham NC. Antibodies used in ELISA and IgG subtype analyses and their respective manufacturers are listed in the Methods. For the SHM sequence and TLR4 expression analyses antibodies used are also listed in the Methods.
Validation	See Methods and references therein.

Eukaryotic cell lines

Policy information about cell lines and Sex and Gender in Research

Cell line source(s)	1: HEK293T/17 (ATCC, cat. CRL-11268)<br>2: TZM-bl. Dr John C. Kappes, Dr. Xiaoyun Wu and Tranzyme Inc. (courtesy of NIH HIV Reagent Program, currently BEI Resources). The cell line was engineered from the a HeLa cell line (human), which was derived from a female donor.
Authentication	1: No authentication was conducted after purchase<br>2: CD4, CCR5, and CXCR4 expression on the cell line was verified.
Mycoplasma contamination	1:Cell line was tested routinely for mycoplasma contamination, and the result was negative.<br>2: Cell line was tested routinely for mycoplasma contamination, and the result was negative.
Commonly misidentified lines (See ICLAC register)	1: No commonly misidentified cell lines were used<br>2: No commonly misidentified cell lines were used

Clinical data

Policy information about clinical studies
All manuscripts should comply with the ICMJE guidelines for publication of clinical research and a completed CONSORT checklist must be included with all submissions.

Clinical trial registration	NCT03961438
Study protocol	Study protocol is accessible through Figshare.
Data collection	Participants made 17 scheduled visits to the Amsterdam UMC, location AMC over the course of 76 weeks, excluding screening. Four additional check-ups were conducted by telephone. Visits were either to the Internal Medicine outpatient clinic, Clinical Experimental Research Unit (vaccination visits), Radiology outpatient clinic (lymph node FNA visits) or the Apheresis unit (leukapheresis visits). Samples were processed the same day and stored at local storage facilities under predefined conditions. Samples were shipped to varying expert laboratories for downstream analyses, if not performed in-house. Safety data was collected by clinical research staff (either nurse or physician) and logged into the electronic participant dossier, before transfer to the electronic Castor EDC database.
Outcomes	Primary and secondary outcomes were pre-defined in the Study Protocol (Section 8.1), in addition to exploratory study outcomes. Outcomes were based on other phase 1 and 2 clinical (HIV) vaccine studies, as well as in house and collaborating expertise regarding (B cell) immunology.

Flow Cytometry

Plots

Confirm that:

- [x] The axis labels state the marker and fluorochrome used (e.g. CD4-FITC).
- [x] The axis scales are clearly visible. Include numbers along axes only for bottom left plot of group (a 'group' is an analysis of identical markers).
- [x] All plots are contour plots with outliers or pseudocolor plots.
- [x] A numerical value for number of cells or percentage (with statistics) is provided.

Methodology

Sample preparation	PBMCs were obtained from fresh blood from a large blood draw or leukapheresis depending on the time point. PBMCs were isolated from blood and frozen at -80 degrees Celcius. Cells were thawed and prepared for sorting on the day of the single cell scRNAseq experiment. Cells were first stained with TotalseqC hashtag antibodies (BioLegend) for sample multiplexing. Next, CD3+ cells were depleted using a CD3 enrichment kit (Stem Cell Technologies). After enrichment cells were stained with ConM SOSIP.v7 dextramer constructs (Immudex), followed by staining with a flow cytometry antibody panel for B-cell phenotyping.
Instrument	BD FACS Symphony S6 sorter 4 laser and BD FACS Aria IIu SORP 4 laser
Software	FlowJo was used to analyse flow cytometry data.
Cell population abundance	Lymphocyte and B cell frequencies in the pre-sort samples were as expected. ConM SOSIP.v7 specific B cells as well as unspecific B cells were sorted for single cell scRNAseq. The proportion and count of vaccine specific and unspecific cells was similar after single cell scRNAseq.
Gating strategy	Lymphocytes are selected from the FSC/SSC plot. To select single cells FSC height and area were used. Live B cells were selected from the single cell population by selecting the CD19 positive and CD4/CD14/CD16/viability negative population. Next, switched and unswitched memory B cells were identified by selecting the IgD positive/CD27 positive and IgD-/CD27 positive populations were selected. In this population, vaccine specific cells were defined as ConM SOSIP.v7 double positive cells.

- [x] Tick this box to confirm that a figure exemplifying the gating strategy is provided in the Supplementary Information.
