# Supplementary material for: HIV-1 envelope trimer vaccine induces sex-associated differences in antibody responses: a phase 1 clinical trial
Source: Nat Commun. 2025 Nov 21;16:10250. doi: 10.1038/s41467-025-65101-7 (PMC12639139; doi:10.1038/s41467-025-65101-7)
Supplement: Supplementary file 3 — Transparent Peer Review file [file 41467_2025_65101_MOESM3_ESM.pdf]

nature portfolio

Peer Review File

HIV-1 envelope trimer vaccine induces sex-associated differences in antibody responses: a phase 1 clinical trial

Corresponding Author: Ms Emma Reiss

This manuscript has been previously reviewed at another journal. This document only contains information relating to versions considered at Nature Communications.

This file contains all reviewer reports in order by version, followed by all author rebuttals in order by version.

Version 0:

Reviewer comments:

Reviewer #1

(Remarks to the Author)
The authors have addressed all my comments. however, I still think the title of the paper seems too bold and conclusive, where the evidence doesn't really support so. will suggest tuning it down.

Reviewer #3

(Remarks to the Author)

Open Access This Peer Review File is licensed under a Creative Commons Attribution 4.0 International License, which permits use, sharing, adaptation, distribution and reproduction in any medium or format, as long as you give appropriate credit to the original author(s) and the source, provide a link to the Creative Commons license, and indicate if changes were

made.
In cases where reviewers are anonymous, credit should be given to 'Anonymous Referee' and the source.
The images or other third party material in this Peer Review File are included in the article’s Creative Commons license, unless indicated otherwise in a credit line to the material. If material is not included in the article’s Creative Commons license and your intended use is not permitted by statutory regulation or exceeds the permitted use, you will need to obtain permission directly from the copyright holder.
To view a copy of this license, visit https://creativecommons.org/licenses/by/4.0/

Reviewer #1 (Remarks to the Author):

The authors have addressed all my comments. however, I still think the title of the paper seems too bold and conclusive, where the evidence doesn't really support so. will suggest tuning it down.

Response: we appreciate the reviewer's comment and have adapted the title.
